# An investigation of biomarkers derived from legacy microarray data for their utility in the RNA-seq era

**DOI:** 10.1186/s13059-014-0523-y

**Published:** 2014-12-03

**Authors:** Zhenqiang Su, Hong Fang, Huixiao Hong, Leming Shi, Wenqian Zhang, Wenwei Zhang, Yanyan Zhang, Zirui Dong, Lee J Lancashire, Marina Bessarabova, Xi Yang, Baitang Ning, Binsheng Gong, Joe Meehan, Joshua Xu, Weigong Ge, Roger Perkins, Matthias Fischer, Weida Tong

**Affiliations:** National Center for Toxicological Research, US Food and Drug Administration, 3900 NCTR Road, Jefferson, AR 72079 USA; Thomson Reuters, IP & Science, 22 Thomson Place, Boston, MA 02210 USA; State Key Laboratory of Genetic Engineering and MOE Key Laboratory of Contemporary Anthropology, Schools of Life Sciences and Pharmacy, Fudan University, Shanghai, 201203 China; Fudan-Zhangjiang Center for Clinical Genomics, Shanghai, 201203 China; Zhanjiang Center for Translational Medicine, Shanghai, 201203 China; BGI-Shenzhen, Main Building, Bei Shan Industrial Zone, Yantian District, Shenzhen, Guangdong 518083 China; BGI-Guangzhou, Guangzhou, China; Department of Pediatric Oncology and Hematology and Center for Molecular Medicine (CMMC), University Children’s Hospital of Cologne, Kerpener Strasse 62, D-50924 Cologne, Germany

## Abstract

**Background:**

Gene expression microarray has been the primary biomarker platform ubiquitously applied in biomedical research, resulting in enormous data, predictive models, and biomarkers accrued. Recently, RNA-seq has looked likely to replace microarrays, but there will be a period where both technologies co-exist. This raises two important questions: Can microarray-based models and biomarkers be directly applied to RNA-seq data? Can future RNA-seq-based predictive models and biomarkers be applied to microarray data to leverage past investment?

**Results:**

We systematically evaluated the transferability of predictive models and signature genes between microarray and RNA-seq using two large clinical data sets. The complexity of cross-platform sequence correspondence was considered in the analysis and examined using three human and two rat data sets, and three levels of mapping complexity were revealed. Three algorithms representing different modeling complexity were applied to the three levels of mappings for each of the eight binary endpoints and Cox regression was used to model survival times with expression data. In total, 240,096 predictive models were examined.

**Conclusions:**

Signature genes of predictive models are reciprocally transferable between microarray and RNA-seq data for model development, and microarray-based models can accurately predict RNA-seq-profiled samples; while RNA-seq-based models are less accurate in predicting microarray-profiled samples and are affected both by the choice of modeling algorithm and the gene mapping complexity. The results suggest continued usefulness of legacy microarray data and established microarray biomarkers and predictive models in the forthcoming RNA-seq era.

**Electronic supplementary material:**

The online version of this article (doi:10.1186/s13059-014-0523-y) contains supplementary material, which is available to authorized users.

## Background

Microarray-based gene expression profiling represents a mature, high-throughput, transcriptomic analysis approach that has been extensively applied in biomedical and clinical research as the major biomarker tool for almost two decades. An important outcome is a number of large-scale microarray data sets for public reference, for example, the Connectivity Map (also known as CMAP) database [1,2], Chemical Effects in Biological Systems (CEBS) [3], DrugMatrix [4], and the Japanese Toxicogenomics Database (TG-GATEs) [5]. Meanwhile, a large number of microarray-based gene signatures and biomarkers [6-9] and gene expression profile-based predictive models [10-12] have also been established for human disease subtype classification, disease diagnosis and prognosis, and therapeutic treatment selection.

During more recent years, next-generation sequencing technologies (NGS) have emerged as a powerful alternative to microarrays, particularly for whole transcriptome analysis with RNA-Seq [13-15]. Besides providing accurate measurement of gene expression levels, RNA-Seq is additionally promising because of its capability to discover splicing junctions, novel transcripts, alternative splicing variants, and un-annotated genes. The unprecedented discovery features as well as a sustained cost decrease are causing an inevitable transition from microarray to RNA-Seq for clinical biomarker development. The advent of evermore economical NGS has led many companies and institutions that have heavily invested in microarrays to ask whether they need to repeat their sample profiling with NGS. Such a costly undertaking could be averted depending on the extent to which predictive models and associated signature genes developed from microarrays can be directly transferred to RNA-Seq data. Given the fact that the cost for RNA-Seq is rapidly decreasing, the same transferability question could be raised again in the future on how predictive models and associated signature genes based on RNA-Seq can be applied back to the legacy microarray data to leverage the existing data and knowledge. Moreover, the analysis of the current Gene Expression Omnibus (GEO) database revealed several important observations (Additional file 1: Figure S1). First, by only examining the number of data added to GEO from both technologies in 2014, much larger number of array data (54,206) was deposited compared to RNA-Seq (9,082). Second, justifying the year as a starting point for which both array data (2001) and RNA-Seq data (2006) were ‘seen’ by GEO, the growth rate for RNA-Seq was slower compared to microarrays in the following 5 to 7 years. Third, projecting the data growth by fitting the existing data with the polynomial and power equations for microarray and RNA-Seq, respectively, it seems that RNA-Seq will reach 1 million mark in 2021 (the current number of arrays in GEO) and surpass microarrays in 2028. The analysis indicated a long period of co-existence of both technologies (the transition from microarray to RNA-Seq could last many years), rendering these aforementioned questions even more important.

As a part of the FDA-led community wide Sequencing Quality Control (SEQC) project [16], we broadly assessed the transferability of predictive models and signature genes between microarray and RNA-Seq data using two large clinical data sets: the neuroblastoma (NB) data (Zhang W, Shi L, Hertwig F, Thierry-Mieg J, Zhang W, Thierry-Mieg D, Wang J, Furlanello C, Devanarayan V, Cheng J, Deng Y, Hero B, Hong H, Jia M, Li L, Lin S, Nikolsky Y, Oberthuer A, Qing T, Su Z, Volland R, Wang W, Wang M, Yu Y, Ai J, Albanese D, Amur S, Asgharzadeh S, Avigad S, Bao W, et al. Comparison of RNA-seq and microarray-based models for clinical endpoint prediction; submitted) having 498 NB samples with six binary clinical endpoints and four continuous survival times and the acute myeloid leukemia (AML) data [17] containing 175 AML samples with two binary clinical endpoints and two continuous survival times (Table 1). ‘Signature genes’ of a predictive model are defined as the set of RNA-Seq genes or microarray probes/probe sets used by the predictive model. The samples in both clinical data sets were independently profiled with microarray and Illumina RNA-Seq technologies. To ensure a rigorous comparison, we first investigated the cross-platform sequence correspondence between microarray probes/probe sets and RNA-Seq genes for three human and two rat data sets having both microarray and RNA-Seq data available for the same samples. Consequently, microarray probes/probe sets and RNA-Seq genes were cross mapped and stratified into four mapping groups A, B, C, and D in accordance with sequence correspondence complexity as defined in Table 2. Three predictive modeling algorithms representing different modeling complexity, k-nearest neighbors (k-NN), nearest shrunken centroids (NSC) [18], and support vector machine (SVM) were applied to each of the three mapping groups A, B, and C, and for each of the eight binary clinical endpoints and Cox proportional hazards survival analysis [19] was applied to the six continuous endpoints to model survival times with gene expression data (Table 1). Our analyses indicate that the signature genes of models between microarray and RNA-Seq data are reciprocally transferable for model development, regardless of the degree of clinical endpoint prediction difficulty and the cross-platform gene mapping complexity. More importantly, the models developed from microarray data could be directly used to accurately predict RNA-Seq-profiled samples, as long as microarray and RNA-Seq data were properly transformed. Conversely, the models derived from RNA-Seq data could be directly used to predict microarray-profiled samples, but with more difficulty and lower accuracy.

**Table 1:** **Definition of the clinical endpoints for the 498 SEQC NB samples and the 175 AML samples**

**Endpoint category**	**Data set**	**Total samples(n)**	**Endpoint**	**Training set**			**Validation set**		
				**Samples(n)**	**1**	**0**	**Samples(n)**	**1**	**0**
Binary (1/0)	SEQC NB	498	A_EFS_All (event, yes/no)	249	89	160	249	94	155
			B_OS_All (death, yes/no)	249	51	198	249	54	195
			C_SEX_All (female/male)	249	103	146	249	108	141
		272	D_FAV_All (unfavorable/favorable)	136	45	91	136	46	90
		176	E_EFS_HR (event, yes/no)	86	55	31	90	65	25
			F_OS_HR (death, yes/no)	86	43	43	90	49	41
	AML	175	Sex (female/male)	89	43	46	86	39	47
			Cytogenetic risk (poor/good)	89	72	17	86	70	16
Continuous (time)	SEQC NB	498	A_EFS_All (event, days)	249			249		
			B_OS_All (death, days)	249			249		
		176	E_EFS_HR (event, days)	86			90		
			F_OS_HR (death, days)	86			90		
	AML	175	EFS (event, months)	89			86		
			OS (death, months)	89			86		

EFS: Event-free survival; FAV: Unfavorable/Favorable (class label for extreme disease course); HR: High-risk patients; OS: Overall survival.

**Table 2:** **Gene mapping groups with different sequence correspondence complexity**

**Group**	**Microarray probes/probe sets**	**RNA-Seq genes**	**Concordance**
A	Each probe set can be exclusive mapped to one RNA-Seq gene. It is identical to RNA-Seq A set	Each gene can be exclusively mapped to one array probe set. It is identical to array A set	High
B	Each probe set can be mapped to one RNA-Seq gene, but the gene can be mapped to multiple array probe sets. It is a subset RNA-Seq C set	Each gene can be mapped to one array probe set, but the probe set can mapped to multiple genes. It is a subset of array C set	Low
C	Each probe set can be mapped to multiple RNA-Seq genes	Each gene can be mapped to multiple array probe sets	Lowest
D	Probe sets cannot be mapped (unique probe set)	Genes cannot be mapped (unique genes)	Unique features

## Results

**Figure 1:**
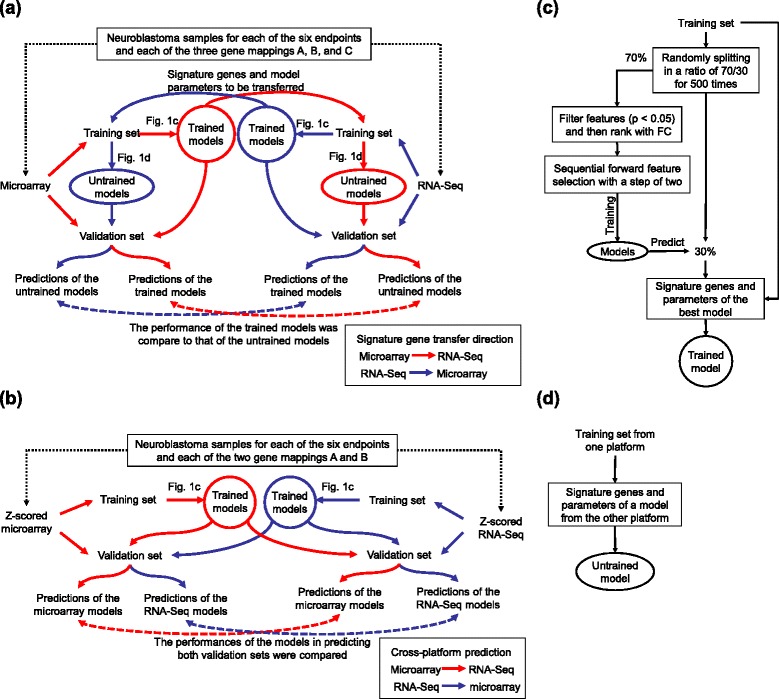
**Flowcharts for evaluating the cross-platform transferability of signature genes and predictive models.** Two analysis procedures were applied to evaluate the transferability of signature genes **(a)** and predictive models **(b)**. In **(a)**, microarray training data are used to develop 500 trained models through **(c)** to predict the microarray validation samples. The signature genes of each model are then used with the RNA-Seq training data to build an untrained RNA-Seq model using through **(d)** to predict the RNA-Seq validation samples. The performance of microarray models is finally compared to that of RNA-Seq models. The transferability of signature genes from RNA-Seq back to microarray data can conversely be calculated. While in **(b)**, both microarray and RNA-Seq data were z-scored prior to model development. Then microarray training data are used to develop 500 trained models to predict both microarray and RNA-Seq validation samples. The performance of models in predicting microarray data is compared to that in predicting RNA-Seq data. From RNA-Seq back to microarray is conversely examined. A trained model is developed through **(c)**. Briefly, training samples are randomly split in a 70/30 ratio. For each split, a series of models are developed using the 70% of training samples to predict the remaining 30%. The models are developed as follows: (1) all genes are first filtered with t-test *P* <0.05 and then ranked by fold change (FC); (2) a sequential forward feature selection by a step of two and parameter selection strategy is then used to build a number of models to predict the remaining samples. Finally, the signature genes and parameters of the best model are used with all training samples to build a trained model. An untrained model is built using all training samples from one platform but with the signature genes and parameters of a model trained from the other platform **(d)**.

**Figure 2:**
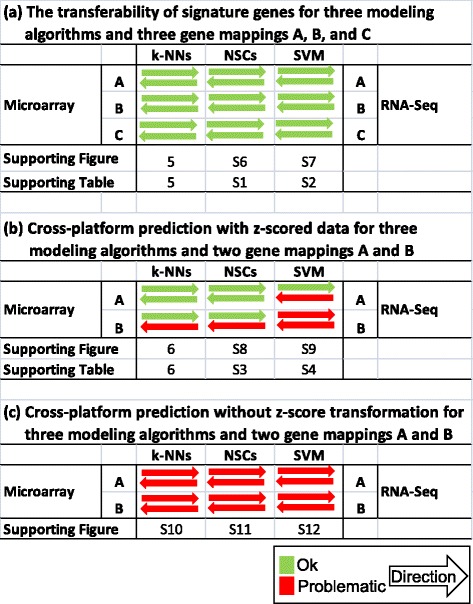
**Summary of the transferability of signature genes and predictive models between microarray and RNA-Seq data.** The test results whether the parameters and signature genes of a model developed from one platform (microarray or RNA-Seq) can be used to build a model using data generated with the other platform (RNA-Seq or microarray) are shown in **(a)** for the three gene mappings A, B, and C separately; while the results whether a predictive model developed from one platform can be directly used to accurately predict the samples profiled with the other platform for gene mappings A and B are summarized for per sample z-scored data and without per sample z-scored data in **(b)** and **(c)**, respectively. Green and red arrows indicate the good and bad transferability from one platform to the other, respectively.

Examining the reciprocal transferability of predictive models and signature genes between microarray and RNA-Seq data requires understanding the sequence correspondence in gene mappings between the two platforms. Consequently, we first performed cross-platform mappings of microarray probes or probe sets to RNA-Seq genes and explored the consistency of gene expression measurements between microarray and RNA-Seq data using two rat and three human data sets having both microarray and RNA-Seq data. The two rat data sets were from the NCTR rat toxicogenomics study [20,21] and the FDA SEquencing Quality Control (SEQC) rat toxicogenomics study [22]; and the three human data sets were from the FDA SEQC main study [16], MicroArray Quality Control (MAQC) phase I main study [23], the SEQC neuroblastoma (NB) study, and The Cancer Genome Atlas (TCGA) acute myeloid leukemia (AML) study [17]. Subsequently, only the FDA SEQC NB and TCGA AML data sets were used for a quantitative assessment of the cross-platform transferability conducted (1) at the signature gene level (Figure 1a) and (2) at the model level [24] (Figure 1b) for both binary endpoint prediction and Cox survival regression analysis. We first applied the whole transferability assessment processes to the FDA SEQC NB data set and then validated the findings using the TCGA AML data set. Since gene mappings between microarray and RNA-Seq are not in one-to-one correspondence, we independently performed the signature level assessment process (Figure 1a) on the three groups of gene mappings (A, B, and C in Table 2) for each of the eight binary predefined clinical endpoints using three modeling algorithms and for each of the six continuous survival times using Cox survival analysis. In total, we carried out 180 signature level assessment processes (three gene mapping groups by eight endpoints by three algorithms by two transfer directions for binary endpoint prediction and three gene mappings by six continuous survival times by two transfer directions for Cox modeling) and thereby generated 144,072 predictive models (500 trained models plus 500 corresponding transferred models per process for binary endpoint prediction and two models per process for Cox modeling). For model level assessment, the process (Figure 1b) was conducted on both original log_2_ intensity/counts data and per sample z-scored data; and group C mappings were excluded from modeling since their ambiguous mapping relationships were not suitable for cross-platform prediction. Thus, we conducted 216 model level assessment processes (two gene mapping groups by eight endpoints by three algorithms by two transfer directions by two forms of data for binary endpoint prediction and two gene mappings by six continuous survival times by two transfer directions for Cox modeling) and thereby generated 96,024 predictive models (500 trained models per process for binary endpoint prediction and one model per process for Cox modeling). Figure 2a,b, and c show the summary of the assessment results for each scenario.

### Cross-platform gene mapping complexity and consistency of gene expression measurements between microarray and RNA-Seq

Gene mapping is an essential step to assess the transferability of gene expression-based predictive models between microarray and RNA-Seq. Cross-platform gene mapping is complicated by disparity between array annotations and RNA-Seq gene models. Moreover, given the complexity of human transcriptomes and the lack of complete genome functional annotation [25], gene annotations from different sources may be inconsistent. Even within a given source, gene annotations undergo constant change. The inconsistency complicates array annotation and causes variation in RNA-Seq analysis as well. We used diverse data sets from microarray and RNA-Seq platforms including Affymetrix rat and human arrays, Agilent human arrays, and Illumina HiSeq 2000, HiScanSQ, and GA-II to characterize and categorize the complexity of cross-platform gene mappings and the consistency of gene expression measurements.

**Figure 3:**
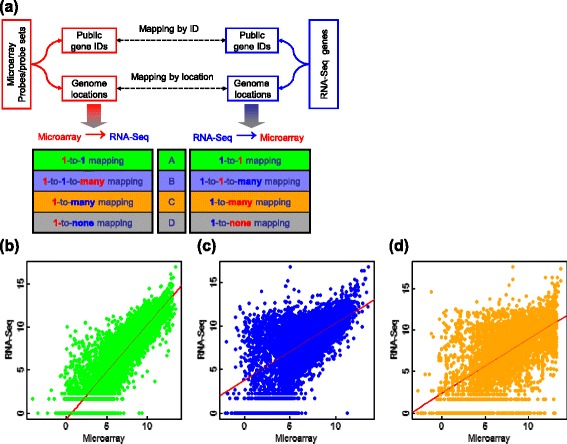
**The strategy for cross-platform gene mapping and the consistency of cross-platform gene expression measurements.** The microarray probes/probe sets are mapped to RNA-Seq genes in one of two ways: public gene ID mapping or genome location mapping **(a)**. Using the gene ID mapping approach requires that one of the following public gene IDs be available: gene symbol, RefSeq transcript ID, Ensembl gene ID, or Entrez gene ID. Using the genome location mapping requires an RNA-Seq gene annotation file in either the Gene Transfer Format (GTF) or the General Feature Format (GFF). The process produces separate mapping lists for microarrays and RNA-Seq. Each of them consists of A, B, C, and D groups. Group A for microarrays corresponds to the group A in RNA-Seq. The microarray group B is a subset of RNA-Seq group C, and *vice versa*. The D group for microarrays and for RNA-Seq contain genes and probes/probe sets that cannot be mapped between the two platforms. The intensities of Affymetrix microarray probe sets in mapping groups A, B, and C are separately compared to those of RNA-Seq gene counts in panels **(b)**, **(c)**, and **(d)** for one of the eight RNA samples in the NCTR toxicogenomics data set. The microarray data are from Rat_230_2 arrays normalized with the MAS5 algorithm, and the RNA-Seq reads are from the Illumina GA II platform with the single-end 36 base pairs RNA-Seq protocol and gene counts from the P2 pipeline (Novoalign with RefSeq rat gene models). The mappings from microarray probe sets to RNA-Seq genes are based on the genome location mapping approach.

We first mapped Affymetrix Rat_230_2 arrays to Illumina GA II RNA-Seq using the method depicted in Figure 3a (see also Methods and Materials). Data were from an NCTR rat toxicogenomics study [20] in which eight rat kidney samples were separately profiled with Rat_230_2 arrays and GA-II RNA-Seq. After mapping array probe sets to RNA-Seq genes, the 31,099 array probe sets were split into four groups A, B, C, and D (defined in Table 2) having 8,350, 7,736, 2,121, and 12,892 probe sets, respectively. Group A contains probe sets that can be exclusively mapped to one RNA-Seq gene; group B includes probe sets that can be uniquely mapped to one RNA-Seq gene, but the RNA-Seq gene can be mapped to multiple array probe sets; group C consists of probe sets that can be mapped to multiple RNA-Seq genes; and group D are microarray unique probe sets that cannot be mapped to any RNA-Seq genes. The gene expression levels in groups A, B, and C detected with microarrays were compared to those detected with RNA-Seq for one of the eight RNA samples (Figure 3b,c, and d). As can be seen from the scatter plots, gene expression measurements for genes in group A are much more consistent than those in groups B and C. The average Spearman’s correlation coefficients between microarray and RNA-Seq measurements for groups A, B, and C are 0.87, 0.60, and 0.54, respectively. The same trend was observed in the mapping results from Affymetrix HG-U133_Plus_2 arrays to Illumina HiSeq 2000 (Table 3 and Additional file 2: Figure S2 and Additional file 3: Figure S3) and from Agilent human arrays to Illumina HiSeq 2000 (Table 3 and Additional file 4: Figure S4).

**Table 3:** **Spearman’s correlation coefficients of gene expression levels detected with microarray and RNA-Seq**

**Data set**	**Microarray**	**RNA-Seq platform and pipeline**	**Gene group**	**Probes/probe sets (n)**	**Spearman’s correlation coefficient**
NCTR rat TGx	Affymetrix	P2	A	8,350	0.87 ± 0.02
	Rat_230_2		B	7,736	0.60 ± 0.04
			C	2,121	0.54 ± 0.04
SEQC main	Affymetrix	P2	A	6,355	0.84 ± 0.01
	HG_U133_Plus 2		B	27,166	0.53 ± 0.02
			C	7,636	0.33 ± 0.02
SEQC NB	Agilent	P2	A	10,042	0.73 ± 0.03
	customized 4 × 44 k		B	13,401	056 ± 0.04
			C	2,543	0.49 ± 0.04
SEQC rat TGx	Affymetrix	P1	A	7,829	0.88 ± 0.01
	Rat_230_2		B	8,376	0.63 ± 0.01
			C	95	0.64 ± 0.03
		P2	A	8,386	0.87 ± 0.01
			B	8,035	0.68 ± 0.01
			C	3,149	0.58 ± 0.01
		P3	A	8,201	0.89 ± 0.01
			B	8,142	0.69 ± 0.01
			C	702	0.73 ± 0.01
		P4	A	8,080	0.88 ± 0.01
			B	8,160	0.67 ± 0.01
			C	91	0.64 ± 0.04
		P5	A	8,197	0.85 ± 0.01
			B	7,543	0.66 ± 0.01
			C	1,750	0.32 ± 0.01
		P6	A	5,663	0.90 ± 0.01
			B	12,228	0.59 ± 0.01
			C	3,189	0.38 ± 0.02
TCCGA ALM	Affymetrix	Bwa + In	A	7,448	0.82 ± 0.04
	HG_U133_Plus 2	House	B	31,313	0.66 ± 0.04
		Program	C	551	0.57 ± 0.04

**Figure 4:**
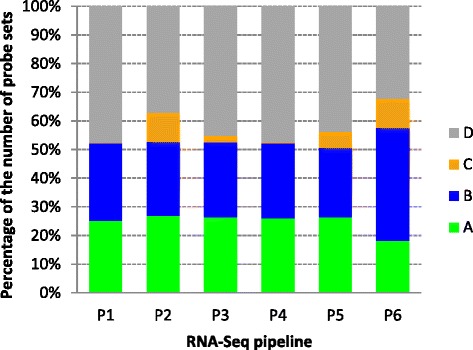
**The percentages of probe sets in mapping groups A, B, C, and D.** The percentages of Affymetrix probe sets in four mapping groups A, B, C, and D for the six RNA-Seq gene sets are shown in stacked bar charts. The data set comprises 62 Affymetrix Rat_230_2 arrays and 62 RNA-Seq assays from the same set of 62 rat liver RNA samples. The microarray data were normalized with MAS5, and the same RNA-Seq raw data were analyzed by six independent data analysis teams with a variety of analysis pipelines, that is, P1 (NCBI Magic), P2 (Novoalign with RefSeq gene models), P3 (Bwa + RefSeq RNAs), P4 (Tophat + HTSeq with RefSeq gene models), P5 (Bowtie + RSEM with Ensembl gene models), and P6 (Tophat + cufflinks *de novo* assembly). The Affymetrix probe sets (31,099 in total) were separately mapped to the six RNA-Seq gene sets. The mappings to P1, P2, P3, and P4 gene sets are based on the gene ID mapping approach, while mappings to P5 and P6 gene sets are based on the genome location mapping.

We then mapped Affymetrix Rat_230_2 arrays to six RNA-Seq gene sets generated from the same raw data of 62 rat liver samples using different bioinformatics pipelines and references, representing a diversity of approaches popularly used in RNA-Seq data analysis [22]. Array probe sets were mapped to each of the six gene sets using either the gene ID or genome location mapping approaches (Table 4). The percentage of array probe sets in groups A, B, C, and D for the six gene sets varied with the choice of analysis pipelines and references (Figure 4). A high percentage of array probe sets (group D) in the range of 32% to 48% could not be mapped to any RNA-Seq genes, though this group of array probe sets provides additional information to RNA-Seq analysis. The correlation pattern exhibited by groups A, B, and C (Table 3 and Additional file 5: Figure S5) is similar to that observed from the previous four analyses. The average Spearman’s correlation coefficients between microarray and RNA-Seq measurements from the 62 rat liver samples for groups A, B, and C are 0.88, 0.65, and 0.55, respectively (Table 3). The results indicate that, although the numbers of genes in the four groups from the six mapping results are quite different (in the range of 5,653 to 8,356, 7,543 to 12,228, 91 to 3,189, and 10,029 to 14,799 for groups A, B, C, and D, respectively), the genes in group A consistently show the highest cross-platform concordance followed by groups B and C. The inconsistency and ambiguity between microarray and RNA-Seq gene models are apparent. In the subsequent analysis, we investigated the cross-platform transferability of signature genes and models separately and explored the potential impact of such gene mapping inconsistency and ambiguity.

**Table 4:** **Bioinformatics pipelines and gene models used for RNA-Seq data analysis**

**Data set**	**Pipeline**	**Aligner**	**Counting and normalization**	**Reference genome**	**Gene annotation**	**Mapping approach**
SEQC NB	P2	Novo align v2.08.01	Global scaling to RPM	UCSC hg19	Human RefSeq RNA v51	ID mapping
NCTR rat toxicogenomics [20]	P2	Novo align v2.08.01	Global scaling to RPM	UCSC rn4	Rat RefSeq RNA v52	Location mapping
SEQC main [16]	P2	Novo align v1.7.01	Global scaling to RPM	UCSC hg19	Human RefSeq RNA v51	Location mapping
SEQC rat toxicogenomics [22]	P1	Magic	Magic index	RGSC v3.4	Ace View 2008 gene models	ID mapping
	P2	Novo align v2.08.01	Global scaling to RPM	UCSC rn4	Rat RefSeq RNA v52	ID mapping
	P3	Bwa 0.5.9-r16	Samtools 0.1.13		Rat RefSeq RNA v50	ID mapping
	P4	To phat	HTSeq-count 0.53p3	UCSC rn4	Rat RefSeq RNA v50	ID mapping
	P5	Bowtie v0.12.7	RSEM v1.1.18	Ensemble rat genome 66	Ensemble genes build 66	Location mapping
	P6	To phat 2.0	Cufflinks + Cuffdiff	UCSC rn4	Cufflinks *de novo* assembly	Location mapping
TCGA AML [17]		Bwa 0.5.7	In-house program	hg18 + exon junction	Ensembl v59	ID mapping

### Signature genes of a microarray model can be transferred to RNA-Seq data for model development

To assess the transferability of signature genes from microarray to RNA-Seq data, we first applied the signature level assessment process (Figure 1a, red line) to the FDA SEQC NB data set. The data set was derived from the 498 NB samples that were independently profiled with Agilent microarrays and Illumina HiSeq 2000. Six binary clinically relevant endpoints of varying degrees of clinical complexity and prediction difficulty were included in the SEQC NB study. As shown in Table 1, A_EFS_All represents event-free or not at a certain time point, where event means the occurrence of progression, relapse or death; B_OS_All denotes whether patients died from disease or not at a specific time point; C_SEX_All is the patients’ sex; D_FAV_All represents event-free without chemotherapy for at least 1,000 days post diagnosis or died from disease despite chemotherapy; E_EFS_HR and F_OS_HR are similar to A_EFS_All and B_OS_All, respectively, but only for the patients with stage four disease or with MYCN-amplified tumors. For each of the six binary endpoints, a set of training samples and a set of validation samples were predefined in the SEQC NB study.

**Figure 5:**
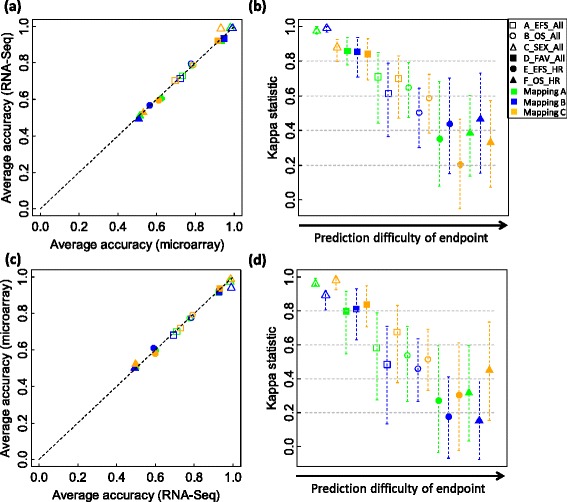
**A performance comparison of k-nearest neighbors (k-NN) models and their corresponding transferred models.** The comparison is based on the SEQC NB data set. For each of the six binary clinical endpoints and each of the three mapping groups A, B, and C, a set of 500 k-NN models were developed from microarray training data and used to predict microarray validation samples. The k parameter and signature genes of each of the 500 microarray models were then used with all RNA-Seq training data for those genes to build an untrained RNA-Seq model to predict RNA-Seq validation samples. Finally, the average prediction accuracies of the 500 microarray models are plotted against those of the 500 corresponding RNA-Seq models **(a)**, with the per sample agreement better than chance given by the Kappa statistic as shown in **(b)**. The transferability of the signature genes from RNA-Seq back to microarray data was conversely calculated. The 500 k-NN models trained from RNA-Seq data were used to predict RNA-Seq validation samples. Then the k parameter and signature genes of each RNA-Seq model were used with all microarray training data for those genes to build a microarray model to predict microarray validation samples. The average accuracies of the 500 RNA-Seq models are compared to those of the 500 corresponding microarray models **(c)**, with the per sample agreement better than chance given by the Kappa statistic as shown in **(d)**. The six symbols in each panel represent the six binary clinical endpoints with green, blue, and orange colors denoting mapping groups A, B, and C, respectively. In panels (b) and (d), each symbol denotes the average Kappa statistic for the 500 pairs of k-NNs models; and each error bar shows the 95% confidence interval (CI) for the mean Kappa statistic. Each CI was calculated with the bootstrap estimation.

We first mapped Agilent array probes to RNA-Seq genes and then sorted them into groups A, B, C, and D in accordance with gene mapping complexity. As indicated above, the probes in groups A, B, and C exhibited different degrees of consistency in gene expression measurements between microarray and RNA-Seq (Table 3 and Additional file 4: Figure S4). We thus separately used the probes in groups A, B, and C as features to develop 500 trained k-nearest neighbors (k-NN) models using microarray training samples for each individual binary clinical endpoint to predict the microarray validation samples. The 500 microarray models were trained using the stratified random sample splitting approach (Figure 1c). For each of the 500 models, the parameter k and signature genes were then used with all RNA-Seq training data for those genes to build an untrained RNA-Seq k-NN model (Figure 1d) to predict the RNA-Seq validation samples. Finally, the prediction performance of the 500 microarray-trained models was compared to that of the 500 corresponding untrained RNA-Seq models to assess the transferability of signature genes from microarray to RNA-Seq. The performance of each model in predicting validation samples was assessed with accuracy and the area under the receiver-operating characteristic curve (AUC) (Table 5). The average prediction accuracies of the microarray models for each mapping group and each endpoint were compared to those of the transferred RNA-Seq models (Figure 5a). All 18 (three mapping groups by six endpoints) average prediction accuracy pairs closely locate to the diagonal of the square indicating the comparable prediction ability of the transferred RNA-Seq models and the original microarray models (paired t-test *P* is 0.718). The comparability is the same in terms of AUC. Per sample agreement beyond chance between the two predictions from each pair of microarray and RNA-Seq models was evaluated with Kappa statistic (Figure 5b). For the endpoints C_SEX_ALL and D_FAV_All which are supposedly easy to predict, model pairs reached perfect agreement (kappa >0.8); for A_EFS_All and B_OS_All, moderate (0.4 < kappa <0.6) to substantial agreement (0.6 < kappa <0.8) was achieved; while for the most difficult to be predicted endpoints E_EFS_HR and F_OS_HR, fair (0.2 < kappa <0.4) to moderate agreement was reached. Therefore, per sample agreement is inversely correlated to the prediction difficulty of the clinical endpoints. The transferability of signature genes from microarray to RNA-Seq data was assessed with T-index score [24]. As shown in Table 5, the T-index scores for transferring signature genes from microarray to RNA-Seq data mainly vary according to the clinical endpoints with only modest variation due to the gene mapping complexity. No significant difference was observed among three mapping groups A, B, and C (one-way ANOVA test *P* is 0.996; pairwise paired t-test *P* values are 0.271, 0.571, and 0.508 for A vs. B, A vs. C, and B vs. C, respectively). Thus, the gene mapping complexity did not affect the transferability of signature genes from microarray to RNA-Seq data.

**Table 5:** **Performance metrics for the assessment of cross-platform transferability of signature genes of k-NN models based on the SEQC NB data**

**Direction**	**Endpoint**	**Gene set**	**Microarray models Is predict microarray validation samples**				**RNA-Seq models predict RNA-Seq validation samples**				**T-index**
			**Accuracy**		**AUC**		**Accuracy**		**AUC**		
			**Mean**	**95% CI**	**Mean**	**95% CI**	**Mean**	**95% CI**	**Mean**	**95% CI**	
From microarray to RNA-Seq	A*	A	0.732	0.667-0.775	0.708	0.645-0.758	0.728	0.653-0.771	0.696	0.627-0.746	0.729
		B	0.721	0.655-0.759	0.691	0.631-0.733	0.714	0.647-0.759	0.678	0.614-0.726	0.716
		C	0.696	0.639-0.735	0.664	0.609-0.704	0.702	0.654-0.747	0.670	0.631-0.713	0.700
	B*	A	0.780	0.747-0.811	0.667	0.615-0.72	0.787	0.755-0.815	0.648	0.601-0.694	0.786
		B	0.777	0.743-0.807	0.643	0.585-0.704	0.794	0.755-0.827	0.659	0.580-0.725	0.791
		C	0.789	0.739-0.827	0.678	0.613-0.732	0.788	0.747-0.819	0.660	0.620-0.711	0.788
	C*	A	0.978	0.971-0.992	0.978	0.971-0.992	0.992	0.992-0.992	0.992	0.992-0.992	0.992
		B	0.989	0.983-0.992	0.989	0.984-0.992	0.989	0.983-0.992	0.989	0.984-0.992	0.989
		C	0.931	0.900-0.954	0.935	0.905-0.958	0.988	0.975-0.992	0.988	0.976-0.992	0.984
	D*	A	0.934	0.882-0.971	0.920	0.863-0.962	0.921	0.882-0956	0.895	0.848-0.940	0.921
		B	0.947	0.824-0.978	0.938	0.798-0.973	0.933	0.772-0.978	0.915	0.737-0.967	0.934
		C	0.915	0.838-0.956	0.911	0.841-0.961	0.921	0.853-0.963	0.914	0.840-0.956	0.920
	E*	A	0.624	0.533-0.700	0.534	0.463-0.615	0.606	0.522-0.689	0.537	0.460-0.617	0.612
		B	0.562	0.478-0.623	0.507	0.417-0.588	0.569	0.500-0.633	0.519	0.443-0.595	0.566
		C	0.607	0.511-0.689	0.513	0.437-0.603	0.599	0.511-0.689	0.515	0.438-0.603	0.602
	F*	A	0.513	0.444-0.589	0.513	0.446-0.585	0.510	0.433-0.589	0.510	0.426-0.589	0.511
		B	0.507	0.456-0.567	0.511	0.452-0.572	0.490	0.422-0.556	0.498	0.431-0.562	0.498
		C	0.532	0.456-0.611	0.534	0.460-0.609	0.527	0.444-0.611	0.531	0.448-0.611	0.529
From RNA-Seq to microarray	A*	A	0.701	0.614-0.759	0.669	0.590-0.732	0.709	0.643-0.759	0.671	0.603-0.727	0.703
		B	0.680	0.584-0.741	0.644	0.543-0.708	0.693	0.604-0.747	0.651	0.568-0.710	0.684
		C	0.719	0.647-0.767	0.691	0.618-0.748	0.730	0.648-0.771	0.698	0.621-0.743	0.722
	B*	A	0.775	0.715-0.819	0.662	0.576-0.737	0.775	0.733-0.811	0.640	0.579-0.696	0.775
		B	0.777	0.715-0.819	0.639	0.552-0.713	0.785	0.735-0.825	0.640	0.566-0.718	0.778
		C	0.792	0.749-0.823	0.681	0.621-0.757	0.790	0.753-0.819	0.659	0.606-0.728	0.791
	C*	A	0.971	0.967-0.992	0.972	0.969-0.992	0.984	0.983-0.992	0.985	0.984-0.992	0.971
		B	0.939	0.900-0.950	0.943	0.901-0.954	0.990	0.988-0.992	0.990	0.988-0.992	0.939
		C	0.987	0.975-0.992	0.987	0.975-0.992	0.987	0.971-0.992	0.987	0.972-0.992	0.987
	D*	A	0.914	0.809-0.971	0.899	0.792-0.957	0.927	0.868-0.963	0.910	0.841-0.951	0.915
		B	0.918	0.838-0.963	0.907	0.825-0.962	0.928	0.860-0.971	0.908	0.836-0.957	0.918
		C	0.937	0.875-0.978	0.923	0.853-0.973	0.933	0.882-0.971	0.913	0.882-0.962	0.937
	E*	A	0.598	0.456-0.689	0.506	0.414-0.606	0.598	0.444-0.700	0.522	0.400-0.630	0.598
		B	0612	0.511-0.700	0.513	0.432-0.602	0.588	0.489-0.678	0.510	0.415-0.613	0.603
		C	0.582	0.478-0.667	0.512	0.412-0.633	0.596	0.500-0.689	0.522	0.432-0.615	0.587
	F*	A	0.507	0.433-0.600	0.502	0.421-0.590	0.498	0.400-0.600	0.496	0.406-0.597	0.503
		B	0.500	0.411-0.595	0.503	0.414-0.596	0.493	0.400-0.589	0.503	0.411-0.601	0.496
		C	0.523	0.456-0.600	0.527	0.452-0.602	0.498	0.405-0.578	0.505	0.411-0.59	0.511

A*: A_EFS_All; B*: B_OS_All; C*: C_SEX_All; D*: D_FAV_All; E*: E_EFS_HR; F*: F_OS_HR; AUC: Area under ROC curve; CI: Confidence interval; 95% CI was calculated from the bootstrap estimation. The upper-right and lower-left regions are for the untrained models built using cross-platform transferred signature genes, while the upper-left and lower-right regions are for the models originally trained.

To confirm these findings, we also applied nearest shrunken centroids (NSC) and support vector machine (SVM) modeling algorithms. Using the same comparison workflow, the results from NSC and SVM are similar to those from k-NN (Additional file 6: Figure S6a and S6b, Additional file 7: Figure S7a and S7b and Additional file 8: Table S1 and Additional file 9: Table S2). Comparing the results from different modeling algorithms shows that, for a specific endpoint, different modeling algorithms might perform differently and result in different T-index scores. For instance, the T-index scores of SVM, k-NN, and NSC models for endpoint ‘A_EFS_All’ are 0.676, 0.729, and 0.734, respectively; but the performance of the predictive models developed from microarrays and transferred from microarrays to RNA-Seq using the same modeling algorithm is consistently comparable.

### Signature genes of a RNA-Seq model can be equally transferred back to microarray data for model development

To assess the transferability of signature genes of models developed from RNA-Seq data to microarray data (Figure 1a, blue line) we again used the FDA SEQC NB data, and again applied k-NN, NSC, and SVM algorithms. We first mapped RNA-Seq genes to Agilent array probes and separated RNA-Seq genes into A, B, C, and D groups, as presented in Table 2. For each algorithm and each group of A, B, and C genes, we used RNA-Seq training samples to develop 500 trained models through the process shown in Figure 1c to predict the RNA-Seq validation samples. Then the parameters and signature genes of each model were used with all microarray training samples for those genes to build an untrained microarray model to subsequently predict the microarray validation samples. The prediction performance of the trained RNA-Seq models was compared to that of the corresponding transferred microarray models using the same metrics as above (Table 5, Figure 5c and d, Additional file 6: Figure S6c, Figure S6d, Additional file 7: Figure S7c, and S7d, and Additional file 8: Table S1 and Additional file 9: Table S2). The results show that the performance of transferred untrained microarray models is comparable to that of original trained RNA-Seq models. There is no significant difference between the accuracies of the untrained microarray models and the trained RNA-Seq models (paired t-test *P* values are 0.356, 0.058, and 0.158 for k-NN, NSC, and SVM, respectively). Thus, signature genes identified from RNA-Seq can also be directly transferred back to microarray data for model development without significant loss of predictive accuracy. Again, the cross-platform gene mapping complexity did not affect the transferability of RNA-Seq signature genes back to microarray data.

### Microarray models can accurately predict samples profiled with RNA-Seq

To test whether the predictive models trained from microarray data can directly predict RNA-Seq-profiled samples, we conducted the model level evaluation process (Figure 1b, red line) on gene mappings A and B separately (the genes in group C were excluded from this analysis due to the mapping ambiguity). Because microarray log_2_ intensity data are quite different from RNA-Seq log_2_ counts, both microarray and RNA-Seq data were z-scored prior to the modeling process. To prevent information leakage, z-score transformation was carried out independently for each sample and within each data set.

**Figure 6:**
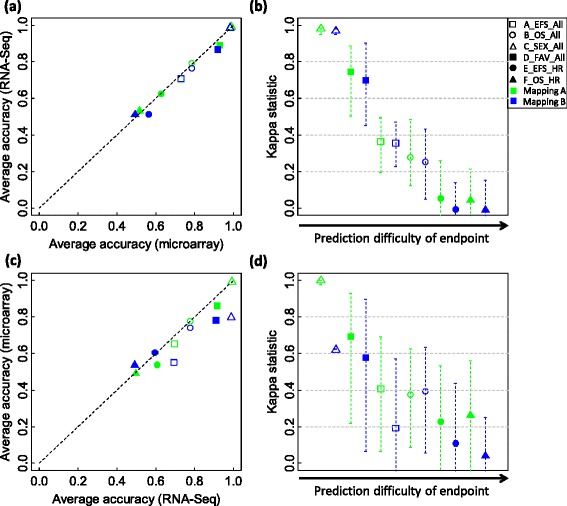
**A performance comparison of k-nearest neighbors (k-NN) in predicting microarray and RNA-Seq validation samples.** The comparison is based on the SEQC NB data set. In the comparison, both microarray log_2_ intensity data and RNA-Seq log_2_ counts were per sample z-scored. For each of the six binary clinical endpoints and each of the two mapping groups A and B, a set of 500 k-NN models were developed from microarray and RNA-Seq training data independently. Each set of k-NN models were then used to predict both microarray and RNA-Seq validation samples. The average prediction accuracies of the 500 microarray k-NN models in predicting microarray data are plotted against those in predicting RNA-Seq data **(a)**, with the per sample agreement better than chance evaluated with the Kappa statistic as shown in **(b)**; while the average accuracies of the 500 RNA-Seq k-NN models in predicting RNA-Seq data are compared to those in predicting microarray data **(c)**, with the per sample agreement better than chance assessed with the Kappa statistic as shown in **(d)**. The six symbols in each panel represent the six binary clinical endpoints with green and blue colors denoting mapping groups A and B, respectively. In panels (b) and (d), each symbol denotes the average Kappa statistic of the 500 pairs of prediction results; and each error bar shows the 95% confidence interval (CI) for the mean Kappa statistic. Each CI was calculated with the bootstrap estimation.

We trained 500 k-NN models from z-scored microarray training data using the approach depicted in Figure 1c and directly applied the models to predict both microarray and RNA-Seq validation samples. The performance of the 500 models in predicting microarray samples in terms of accuracy and AUC was compared to that in predicting RNA-Seq samples (Figure 6a and Table 6). The average accuracies of the models in predicting microarray data are quite close to those in predicting RNA-Seq data (Figure 6a), indicating that the microarray models can directly predict RNA-Seq-profiled samples without significant loss of prediction performance (paired t-test *P* is 0.093). The per sample prediction agreement assessed with the Kappa statistic is inversely correlated to the complexity of the clinical endpoints (Figure 6b). The difference between T-index scores (Table 6) from gene mappings A and B is not significant (paired t-test *P* is 0.106). The results from NSC algorithm are similar to those from k-NN (Additional file 10: Figure S8a and S8b and Additional file 11: Table S3).

**Table 6:** **The performance of k-NN models in predicting microarray and RNA-Seq validation samples based on the SEQC NB data**

**Direction**	**End point**	**Gene set**	**Predicto mg microarray validation data**				**Predicting RNA-Seq validation data**				**T-index**
			**Accuracy**		**AUC**		**Accuracy**		**AUC**		
			**Mean**	**95% CI**	**Mean**	**95% CI**	**Mean**	**95% CI**	**Mean**	**95% CI**	
Models developed from microarray	A*	A	0.728	0.659-0.775	0.698	0.633-0.752	0.706	0.606-0.763	0.678	0.596-0.747	0.712
		B	0.730	0.655-0.775	0.694	0.619-0.749	0.709	0.640-0.755	0.671	0.610-0.730	0.714
	B*	A	0.786	0.751-0.815	0.662	0.600-0.719	0.792	0.743-0.831	0.621	0.547-0.738	0.791
		B	0.785	0.743-0.821	0.638	0.572-0.708	0.764	0.663-0.815	0.621	0.521-0.737	0.768
	C*	A	0.988	0.983-0.992	0.988	0.983-0.992	0.990	0.975-0.992	0.990	0.977-0.992	0.990
		B	0.982	0.971-0.992	0.983	0.974-0.992	0.984	0.975-0.992	0.985	0.976-0.995	0.984
	D*	A	0.928	0.860-0.971	0.907	0.831-0.962	0.890	0.790-0.949	0.860	0.728-0.937	0.891
		B	0.918	0.816-0.982	0.900	0.781-0.978	0.868	0.746-0.956	0.841	0.736-0.945	0.870
	E*	A	0.625	0.522-0.711	0.540	0.446-0.637	0.627	0.456-0.733	0.529	0.440-0.636	0.626
		B	0.564	0.456-0.661	0.489	0.394-0.573	0.516	0.278-0.711	0.497	0.429-0.575	0.533
	F*	A	0.516	0.433-0.600	0.515	0.432-0.597	0.532	0.433-0.622	0.521	0.438-0.607	0.525
		B	0.493	0.422-0.556	0.497	0.430-0.562	0.512	0.422-0.600	0.495	0.415-0.575	0.503
Models developed from RNA-Seq	A*	A	0.655	0.456-0.759	0.653	0.528-0.752	0.695	0.610-0.747	0.656	0.586-0.714	0.665
		B	0.551	0.367-0.719	0.572	0.421-0.705	0.692	0.614-0.747	0.651	0.575-0.710	0.584
	B*	A	0.778	0.715-0.811	0.571	0.516-0.636	0.776	0.735-0.815	0.641	0.583-0.704	0.777
		B	0.740	0.524-0.807	0.643	0.500-0.768	0.775	0.723-0.815	0.629	0.556-0.698	0.746
	C*	A	0.991	0.979-0.992	0.991	0.981-0.992	0.992	0.992-0.992	0.991	0.992-0.992	0.991
		B	0.797	0.797-0.801	0.823	0.822-0.826	0.987	0.979-0.992	0.987	0.981-0.992	0.796
	D*	A	0.862	0.651-0.956	0.832	0.632-0.945	0.914	0.846-0.956	0.896	0.809-0.946	0.863
		B	0.781	0.408-0.941	0.812	0.548-0.937	0.910	0.824-0.956	0.890	0.782-0.946	0.775
	E*	A	0.538	0.333-0.689	0.546	0.452-0.632	0.606	0.478-0.706	0.526	0.432-0.618	0.560
		B	0.607	0.400-0.722	0.502	0.420-0.609	0.593	0.500-0.689	0.511	0.419-0.615	0.603
	F*	A	0.491	0.411-0.584	0.500	0.421-0.588	0.497	0.405-0.589	0.495	0.401-0.588	0.494
		B	0538	0.456-0.600	0.505	0.440-0585	0.492	0.411-0.567	0.502	0.424-0.574	0.516

When SVM algorithm was applied, the difference between the model performances in predicting microarray and RNA-Seq data was quite large (Additional file 12: Figure S9a and S9b and Additional file 13: Table S4), particularly for endpoint C_SEX_All and gene mapping group B (the average accuracy dropped from 0.967 to 0.824). The difference between the average accuracies in predicting microarray and RNA-Seq data is significant (paired t-test *P* is 0.008). Therefore, the transferability of microarray models to predict RNA-Seq data is dependent on the choice of modeling algorithms. For algorithms that are not too sensitive to data values such as k-NN and NSC, microarray-based models can directly be applied for prediction of RNA-Seq-profiled samples; while for data-value-sensitive algorithms such as SVM, such a direct application is challenging.

### It is more difficult to use RNA-Seq models to predict microarray-profiled samples

We next examined the transferability of models developed from RNA-Seq data back to predict microarray data (Figure 1b, blue line). A set of 500 k-NN models were trained from z-scored RNA-Seq training data and used to predict both RNA-Seq and microarray validation samples. As shown in Figure 6c and d and Table 6, the accuracies of the RNA-Seq models developed from mapping group B in predicting microarray validation samples for endpoints A_EFS_All, B_OS_All, C_SEX_All, and D_FAV_All decreased considerably compared to that in predicting RNA-Seq-profiled samples; while the models developed from mapping group A achieved comparable accuracies in prediction both microarray and RNA-Seq validation samples. The similar results were observed with NSC (Additional file 10: Figure S8c and S8d and Additional file 11: Table S3). Thus, the transferability of RNA-Seq models back to predict microarray data can be substantially affected by the lack of cross-platform gene mapping correspondence.

The performance of the RNA-Seq models dropped dramatically in predicting microarray-profiled validation samples when SVM was used, regardless which mapping group of genes were used to develop RNA-Seq models (Additional file 12: Figure S9c and S9d and Additional file 13: Table S4). Clearly, it is more difficult and degraded accuracy should be expected when using the SVM algorithm to develop RNA-Seq models to predict microarray data.

### Data transformation is required to use the models developed from one platform to predict samples profiled with the other platform

We also evaluated the model level transferability (Figure 1b) without z-score preprocessing (that is, using log_2_ intensity and log_2_ counts data for microarray and RNA-Seq, respectively). The accuracies of the models in cross-platform prediction dropped dramatically for most endpoints compared to that in predicting the samples profiled with the same platform as used for model development (Additional file 14: Figure S10, Additional file 15: Figure S11, and Additional file 16: Figure S12). The results suggest that it is essential to adequately transform both microarray and RNA-Seq data prior to model development and cross-platform prediction.

### The transferability of Cox models from survival analysis follow the similar patterns as observed from the binary endpoint prediction analyses

The analyses above only used binary endpoints (A_EFS_All, B_OS_All, E_EFS_HR, and F_OS_HR) for the prediction of patient’s survival status. Because survival times were not considered during modeling process, the analyses may not be extrapolated to the models in which survival times were directly modeled. To examine the transferability of such models and associated signature genes, we applied Cox proportional hazards survival regression to model survival times with gene expression data for the SEQC NB data set and compared the performance of Cox models in terms of concordance index and *P* value calculated with concordance.index and cindex.comp functions from R package survcomp [26]. The concordance index estimates the probability of concordance between predicted and observed responses with values of 0.5, 1, and 0 for random guessing, perfect prediction, and anti-perfect prediction, respectively [27].

To train a Cox’s model, the genes in a training set were first filtered with their median intensities (the median intensity of a gene across the training samples is greater than the median intensity of all genes across all training samples) and *P* values (<0.01) calculated with the function of concordance.index in R package survcomp [26] and ranked according to their concordance indices. The signature genes were then selected by running a leave-one-out cross-validation process with a one-step forward gene selection approach. The final Cox model was built using all training samples with the selected signature genes.

To assess gene level transferability, a Cox model was first trained from a training data set from one platform through the leave-one-out cross-validation process and then used to predict the corresponding validation samples profiled with the same platform. The signature genes were then used with the training data set from the other platform to build a Cox model to predict the corresponding validation samples. The performance of each Cox model was assessed with a concordance index with a *P* value indicating whether the concordance index is significantly different from 0.5. The two concordance indices were finally compared with the function of cindex.comp to test whether the first concordance index is significantly greater than the second. As shown in Additional file 17: Table S5, for the patient cohorts of A_EFS_All and B_OS_All that are easier to predict, the signature genes of Cox models can be easily transferred between the two platforms for Cox model development. But for the high-risk cohorts (E_EFS_HR and F_OS_HR) that are supposedly more difficult to predict, the transferability is much lower. The results are consistent with those from the binary endpoint prediction analyses.

To examine model level transferability, we first did per-sample z-score transformation for data and then trained a Cox model using a training set from one platform and then applied it to separately predict the validation samples profiled with the two platforms. The performance of the model in predicting both validation samples was separately measured with the concordance index and then the two concordance indices were compared to each other with cindex.comp. The transferability of Cox models between microarray and RNA-Seq data sets also shows a similar pattern to those from the previous binary endpoint prediction analyses (Additional file 18: Table S6).

### Validation of the findings using The Cancer Genome Atlas (TCGA) acute myeloid leukemia (AML) data

To validate the findings based on the NB data set, we repeated the same analysis processes to the TCGA AML data set which contains 175 Affymetrix HG-U133_plus_2 microarrays and Illumina HiSeq 2000 RNA-Seq assays from the same set of AML tumor RNA samples (see Methods and Materials) with two binary (sex and cytogenetic risk status) and two continuous (event-free survival time (EFS) and overall survival time (OS)) endpoints. For binary endpoint prediction, the three binary modeling algorithms (that is, k-NN, NSC, and SVM) were separately applied to predict patients’ sex and cytogenetic risk status. While the time to EFS and OS events of patients were modelled with Cox proportional hazards regression as we did for the NB data set.

**Figure 7:**
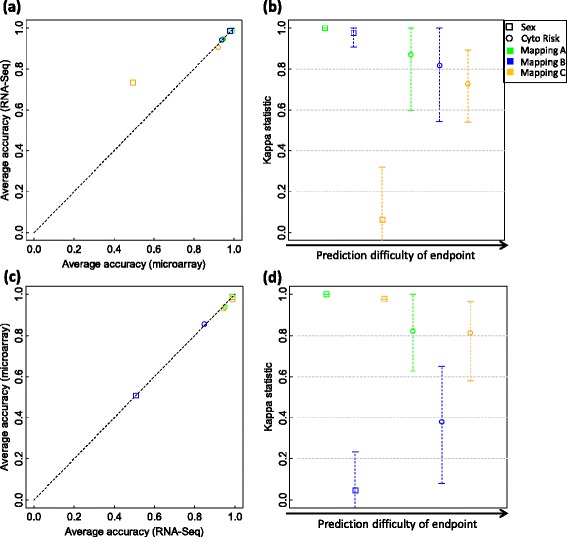
**A performance comparison of k-nearest neighbors (k-NN) models and their corresponding transferred models based on the TCGA AML data.** For each of the two binary clinical endpoints and each of the three mapping groups A, B, and C, a set of 500 k-NN models were developed from microarray training data and used to predict microarray validation samples. The signature genes of each of the 500 microarray models were then used with all RNA-Seq training data for those genes to build an untrained RNA-Seq model to predict RNA-Seq validation samples. Finally, the average prediction accuracies of the 500 microarray models are plotted against those of the 500 corresponding RNA-Seq models **(a)**, with the per sample agreement better than chance evaluated with the Kappa statistic as shown in **(b)**. The transferability of the signature genes from RNA-Seq back to microarray data was conversely calculated. The 500 k-NN models trained from RNA-Seq data were used to predict RNA-Seq validation samples. Then the signature genes of each RNA-Seq model were used with all microarray training data for those genes to build an untrained k-NN model to predict microarray validation samples. The average accuracies of the 500 RNA-Seq models were then compared to those of the 500 corresponding microarray models **(c)**, with the per sample agreement better than chance assessed with the Kappa statistic as shown in **(d)**. The two symbols in each panel represent the two binary clinical endpoints with green, blue, and orange colors denoting mapping groups A, B, and C, respectively. In panels **(b)** and **(d)**, each symbol denotes the average Kappa statistic of the 500 pairs of model predictions; and each error bar shows the 95% confidence interval (CI) for the mean Kappa statistic. Each CI was calculated with the bootstrap estimation. No significant difference is observed between trained microarrays models and transferred RNA-Seq models (paired t-test *P* is 0.366) and between the trained RNA-Seq models and the transferred microarray models (paired t-test *P* is 0.269).

At signature gene level, the untrained RNA-Seq models built with the signature genes of trained microarray models show comparable prediction performance except for using the mapping group C to predict the sex of patients (Figure 7a and b and Table 7; Additional file 19: Figure S13a and S13b; Additional file 20: Figure S14a and S14b). Using mapping group C, microarray-based trained models cannot accurately predict the sex endpoint (about 50% accuracy). However, the transferred RNA-Seq models show much better prediction capability (about 73% accuracy). This could be explained by the more accurate measurements of RNA-Seq. The microarray models built with the signature genes of trained RNA-Seq models consistently show comparable prediction performance compared to that of trained RNA-Seq models (Figure 7c and d and Table 7, Additional file 19: Figure S13c and S13d; Additional file 20: Figure S14c and S14d). For EFS and OS survival time Cox regression analysis, the original trained models and transferred models did not show significant difference (Additional file 21: Table S7, p4 > 0.01) except for using mapping group C to predict EFS time in which the original trained microarray models outperformed the transferred RNA-Seq untrained models (p4 < 0.0018). Therefore, the signature genes are reciprocally transferable between microarray and RNA-Seq data.

**Table 7:** **Performance metrics for the assessment of cross-platform transferability of signature genes of predictive models based on the TCGA AML data**

**Algorithm**	**Direction**	**Endpoint**	**Gene set**	**Microarray model**				**RNA-Seqmodel**				**T-index**
				**Accuracy**		**AUC**		**Accuracy**		**AUC**		
				**Mean**	**95% CI**	**Mean**	**95% CI**	**Mean**	**95% CI**	**Mean**	**95% CI**	
k-NN	Forward	Sex	A	0.988	0.988-0.988	0.987	0.987-0.987	0.988	0.988-0.988	0.987	0.987-0.987	0.988
			B	0.980	0.953-0.988	0.980	0.955-0.987	0.986	0.977-0.988	0.985	0.977-0.987	0.986
			C	0.496	0.419-0.57	0.496	0.413-0.579	0.734	0.419-0.988	0.733	0.418-0.987	0.647
		Cyto Risk	A	0.945	0.907-0.988	0.895	0.805-0.993	0.947	0.872-0.988	0.905	0.794-0.993	0.947
			B	0.939	0.872-0.977	0.904	0.745-0.986	0.942	0.884-0.977	0.925	0.825-0.986	0.942
			C	0.919	0.848-0.965	0.884	0.767-0.964	0.907	0.872-0.953	0.854	0.776-0.948	0.908
	Reverse	Sex	A	0.988	0.988-0.988	0.987	0.987-0.987	0.988	0.988-0.988	0.987	0.987-0.987	0.988
			B	0.508	0.419-0.599	0.501	0.41-0.591	0.506	0.407-0.593	0.508	0.409-0.593	0.507
			C	0.977	0.977-0.977	0.977	0.977-0.977	0.988	0.988-0.988	0.987	0.987-0.987	0.977
		Cyto Risk	A	0.937	0.837-0.988	0.886	0.724-0.993	0.951	0.843-0.988	0.924	0.815-0.993	0.938
			B	0.856	0.744-0.919	0.758	0.59-0.888	0.848	0.767-0.907	0.748	0.589-0.893	0.855
			C	0.930	0.86-0.965	0.887	0.767-0.979	0.943	0.86-0.988	0.920	0.746-0.993	0.930
NSC	Forward	Sex	A	0.988	0.988-0.988	0.987	0.987-0.987	0.988	0.988-0.988	0.987	0.987-0.987	0.988
			B	0.983	0.977-0.988	0.982	0.977-0.987	0.988	0.988-0.988	0.987	0.987-0.987	0.988
			C	0.471	0.419-0.547	0.454	0.398-0.531	0.725	0.488-0.988	0.701	0.46-0.987	0.619
		Cyto Risk	A	0.937	0.907-0.953	0.857	0.75-0.907	0.951	0.919-0.977	0.904	0.836-0.979	0.950
			B	0.892	0.837-0.93	0.860	0.707-0.933	0.891	0.872-0.907	0.873	0.801-0.919	0.891
			C	0.889	0.849-0.907	0.812	0.666-0.871	0.876	0.86-0.919	0.775	0.625-0.829	0.877
	Reverse	Sex	A	0.988	0.988-0.988	0.987	0.987-0.987	0.988	0.988-0.988	0.987	0.987-0.987	0.988
			B	0.526	0.453-0.547	0.487	0.439-0.52	0.504	0.436-0.547	0.481	0.426-0.52	0.515
			C	0.988	0.988-0.988	0.987	0.987-0.987	0.988	0.988-0.988	0.987	0.987-0.987	0.988
		Cyto Risk	A	0.926	0.872-0.977	0.828	0.656-0.986	0.929	0.907-0.977	0.913	0.846-0.986	0.927
			B	0.854	0.814-0.895	0.609	0.5-0.743	0.820	0.814-0.837	0.521	0.5-0.562	0.849
			C	0.907	0.884-0.925	0.907	0.863-0.954	0.886	0.872-0.942	0.852	0.794-0.964	0.905
SVM	Forward	Sex	A	0.980	0.965-0.988	0.980	0.966-0.987	0.988	0.988-0.988	0.987	0.987-0.987	0.988
			B	0.981	0.942-0.988	0.981	0.945-0.987	0.984	0.977-0.988	0.983	0.974-0.987	0.984
			C	0.516	0.43-0.593	0.515	0.429-0.599	0.753	0.43-0.988	0.752	0.426-0.987	0.671
		Cyto Risk	A	0.939	0.895-0.988	0.879	0.774-0.993	0.957	0.895-0.988	0.919	0.774-0.993	0.956
			B	0.963	0.919-0.988	0.941	0.829-0.993	0.961	0.93-0.988	0.947	0.868-0.993	0.961
			C	0.919	0.884-0.953	0.901	0.832-0.964	0.920	0.86-0.965	0.888	0.736-0.979	0.920
	Reverse	Sex	A	0.981	0.965-0.988	0.980	0.964-0.987	0.988	0.988-0.988	0.987	0.987-0.987	0.981
			B	0.493	0.407-0.593	0.489	0.404-0.592	0.497	0.407-0.593	0.497	0.409-0.588	0.495
			C	0.982	0.977-0.988	0.982	0.977-0.987	0.988	0.977-0.988	0.987	0.974-0.987	0.983
		Cyto Risk	A	0.932	0.872-0.977	0.871	0.739-0.962	0.965	0.919-0.988	0.955	0.812-0.993	0.932
			B	0.845	0.756-0.919	0.778	0.608-0.895	0.869	0.779-0.942	0.787	0.583-0.925	0.847
			C	0.944	0.872-0.977	0.901	0.729-962	0.964	0.907-0.988	0.944	0.846-0.993	0.944

AUC: Area under ROC curve; CI: Confidence interval; 95% CI was calculated from the bootstrap estimation.

A*: A_EFS_All; B*: B_OS_All; C*: C_SEX_All; D*: D_FAV_All; E*: E_EFS_HR; F*: F_OS_HR; AUC: Area under ROC curve; CI: Confidence interval; 95% CI was calculated from the bootstrap estimation. The upper-right and lower-left regions are for the cross-platform prediction of the models (the training and validation samples were profiled with different platforms), while the upper-left and lower-right regions are for the intra-platform prediction of the models (both training and validation samples were profiled with the same platform).

**Figure 8:**
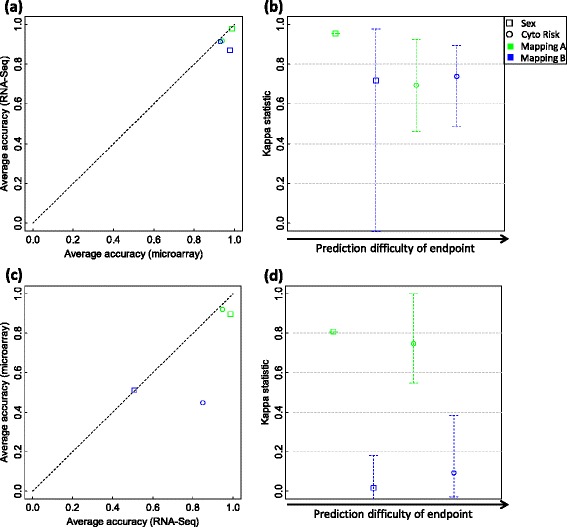
**A performance comparison of k-nearest neighbors (k-NN) models in predicting microarray and RNA-Seq validation data based on the TCGA AML data.** In the comparison, both microarray log_2_ intensity and RNA-Seq log_2_ count were per sample z-scored. For each of the two binary clinical endpoints and each of the two mapping groups A and B, a set of 500 k-NN models were developed from microarray and RNA-Seq training data independently. Each set of k-NN models were then used to predict both microarray and RNA-Seq validation samples. The average prediction accuracies of the 500 microarray-based models in prediction microarray data were plotted against those in predicting RNA-Seq data **(a)**, with per sample agreement better than chance assessed with the Kappa statistic as shown in **(b)**; while the average accuracies of the 500 RNA-Seq-based models in predicting RNA-Seq data were compared to those in predicting microarray data **(c)**, with per sample agreement better than chance evaluated with the Kappa statistic as shown in **(d)**. The two symbols in each panel represent the two binary clinical endpoints with green and blue colors denoting mapping groups A and B, respectively. In panels **(b)** and **(d)**, each symbol denotes the average Kappa statistic of 500 pairs of prediction results; and each error bar shows the 95% confidence interval (CI) for the mean Kappa statistic. Each CI was calculated with the bootstrap estimation.

Using the models developed from one platform to directly predict the samples whose expression value obtained from the other platform, the results were dependent on several factors, that is, the selection of mapping groups, the choice of machine learning algorithms, and/or with or without proper data transformation (Figure 8 and Table 8, Additional file 22: Figure S15, Additional file 23: Figure S16, Additional file 24: Figure S17, Additional file 25: Figure S18, Additional file 26: Figure S19). Specifically, we found that, using microarray-based models to classify samples with RNA-Seq based expression data, (1) k-NN performed well except for mapping group B to classify patients’ sex (Figure 8a and b), (2) NSC worked well for both endpoints (sex and cytogenetic risk status) and for both mapping groups (A and B) (Additional file 22: Figure S15a and S15b), however (3) SVM performed well only for cytogenetic risk status prediction (Additional file 23: Figure S16a and S16b). Conversely, we found that more difficult to classify samples with microarray data using the models developed with RNA-seq data. For example, the performance of some models decreased dramatically (Figure 8c and d and Table 8, Additional file 22: Figure S15c and S15d, Additional file 23: Figure S16c and S16d). When using the original expression data without per sample z-score transformation, the models developed from one platform cannot accurately predict the samples with gene expression data obtained from the other platform (Additional file 24: Figure S17, Additional file 25: Figure S18 and Additional file 26: Figure S19). The EFS and OS survival time Cox regression analysis shows the same trend as from the SEQC NB data (Additional file 27: Table S8).

**Table 8:** **The performance of models developed from one-platform in predicting microarray and RNA-Seq validation samples based on the TCGA AML data**

**Algorithm**	**Direction**	**End point**	**Gene set**	**Microarray data**				**RNA-Seq data**				**T-index**
				**Accuracy**		**AUC**		**Accuracy**		**AUC**		
				**Mean**	**95% CI**	**Mean**	**95% CI**	**Mean**	**95% CI**	**Mean**	**95% CI**	
k-NN	Forward	Sex	A	0.988	0.988-0.988	0.987	0.987-0.987	0.977	0.977-0.977	0.977	0.977-0.977	0.977
			B	0.978	0.942-0.988	0.978	0.945-0.987	0.869	0.523-0.988	0.857	0.481-0.987	0.851
		Cyto Risk	A	0.939	0.895-0.988	0.884	0.805-0.993	0.916	0.837-0.977	0.825	0.68-0.979	0.917
			B	0.931	0.837-0.977	0.890	0.69-0.986	0.914	0.837-0.965	0.903	0.736-0.979	0.914
	Reverse	Sex	A	0.895	0.895-0.895	0.885	0.885-0.885	0.988	0.988-0.988	0.987	0.987-0.987	0.896
			B	0.512	0.419-0.593	0.503	0.426-0.58	0.508	0.407-0.593	0.509	0.409-0.6	0.510
		Cyto Risk	A	0.920	0.866-0.988	0.864	0.736-0.993	0.949	0.837-0.988	0.916	0.822-0.993	0.921
			B	0.447	0.186-0.849	0.609	0.5-0.802	0.850	0.767-0.919	0.747	0.587-0.896	0.425
NSC	Forward	Sex	A	0.988	0.988-0.988	0.987	0.987-0.987	0.978	0.977-0.988	0.978	0.977-0.987	0.978
			B	0.982	0.977-0.988	0.981	0.977-0.987	0.988	0.988-0.988	0.987	0.987-0.987	0.988
		Cyto Risk	A	0.931	0.872-0.953	0.840	0.656-0.899	0.927	0.86-0.977	0.828	0.649-0.962	0.927
			B	0.884	0.826-0.919	0.843	0.676-0.923	0.888	0.872-0.892	0.885	0.815-0.912	0.888
	Reverse	Sex	A	0.977	0.977-0.977	0.974	0.974-0.974	0.988	0.988-0.988	0.987	0.987-0.987	0.977
			B	0.523	0.442-0.558	0.502	0.472-0.553	0.508	0.442-0.558	0.486	0.438-0.539	0.516
		Cyto Risk	A	0.906	0.837-0.965	0.788	0.635-0.947	0.924	0.895-0.953	0.893	0.839-0.968	0.907
			B	0.663	0.198-0.919	0.704	0.5-0.854	0.819	0.814-0.837	0.520	0.5-0.579	0.653
SVM	Forward	Sex	A	0.984	0.977-0.988	0.983	0.977-0.987	0.713	0.453-0.988	0.737	0.5-0.987	0.641
			B	0.981	0.93-0.988	0.981	0.934-0.987	0.817	0.488-0.988	0.799	0.45-0.987	0.782
		Cyto Risk	A	0.925	0.86-0.965	0.849	0.736-0.93	0.909	0.837-0.988	0.787	0.611-0.993	0.910
			B	0.950	0.831-0.977	0.916	0.66-0.986	0.935	0.849-0.977	0.924	0.704-0.986	0.935
	Reverse	Sex	A	0.603	0.547-0.698	0.563	0.5-0.667	0.986	0.977-0.988	0.985	0.974-0.987	0.587
			B	0.510	0.453-0.547	0.500	0.458-0.549	0.500	0.407-0.599	0.499	0.401-0.6	0.505
		Cyto Risk	A	0.897	0.826-0.977	0.777	0.562-0.979	0.961	0.924-0.988	0.945	0.837-0.993	0.896
			B	0.789	0.535-0.814	0.487	0.339-0.5	0.874	0.767-0942	0.798	0.61-0.94	0.793

AUC: Area under ROC curve; CI: Confidence interval; 95% CI was calculated from the bootstrap estimation.

In summary, the results from the analysis of the TCGA AML data are consistent with those from the analysis of the SEQC NB data.

## Discussion

As a part of SEQC project, we used two large clinical data sets to comprehensively investigate the transferability of predictive models and associated signature genes derived from microarrays to RNA-Seq data, and reciprocally from RNA-Seq back to microarray data. The study design is comprehensive. First, since the nature of an endpoint is the most significant factor to determine the robustness of a predictive model [28], our study contains eight binary endpoints and six continuous survival times with varying degrees of difficulty for prediction (Table 1). Second, we observed large variation derived from array annotations and RNA-Seq gene models when mapping genes between microarray and RNA-Seq (Figures 3b,c,d, and 4). Therefore, the cross-platform gene mapping complexity was taken into consideration in the analysis. Third, realizing the choice of modeling algorithms could affect observations; three representative classification algorithms from simple to complicated were selected according to their mathematical complexity. Last and most importantly, the investigation was conducted at both signature gene (Figure 1a) and model levels (Figure 1b) and the conclusions were drawn from the prediction of external validation data sets. With this design, several important observations were made (Figure 2a,b and c).

We observed that signature genes derived from one platform can be directly used to build predictive models using data generated from the other platform. We also demonstrated that microarray-base predictive models can directly predict RNA-Seq-profiled samples, but the reverse process yielded less accuracy. Apparently, the effect of the cross-platform gene mapping complexity was minimal to the transferability of signature genes between the two platforms, but did show influence to a certain degree at the model level. This could result from the model ‘recalibration’ step (Figure 1d) at signature level transfer. The ‘recalibration’ might take care of the cross-platform discordance at absolute gene expression measurement. In addition, neither k-NN nor NSC models developed from microarray data were detrimentally affected by the cross-platform gene mapping ambiguity in predicting RNA-Seq-profiled samples. However, both k-NN and NSC models developed from RNA-Seq data using mapping group B were unfavorably affected by the cross-platform gene mapping ambiguity.

The choice of modeling algorithms was also found to affect the goodness of model level transferability. Because the SVM algorithm is much more sensitive than k-NN and NSC to data values, SVM models developed from one platform in predicting samples profiled with the other platform suffered large degradation of accuracy compared to those in predicting samples profiled with the same platform. Relatively, the models developed using k-NN and NSC algorithms were much more robust than SVM.

Because of the systematic differences between microarray and RNA-Seq gene expression measurements, proper data transformation is essential to develop a predictive model for the cross-platform prediction. Our analysis demonstrated that per sample z-score transformation is such an adequate approach, and furthermore has no leakage of information from validation samples to training process.

Microarray annotations are subject to constant updating with changes from RefSeq, GenBank, and Ensembl databases. RNA-Seq gene models also vary with improving knowledge about the genome and functional elements [29]. Such changes increase the complexity of cross-platform gene mappings. Previous studies suggest that the changes of array annotations can induce variability in comparisons of different microarray technologies [23,30]. The current study found that array annotations and RNA-Seq gene models can cause variation too when comparing gene expression levels from microarrays and RNA-Seq. The finding might provide a partial explanation as to why the overlap of differentially expressed genes from microarrays and RNA-Seq is quite low [20].

We learned from the FDA-led second phase of MicroArray Quality Control (MAQC-II) study that the prediction performance of microarray gene expression profile-based models is mainly dependent on endpoints [28]. The results of this study indicate that the transferability of predictive models and associated signature genes between microarray and RNA-Seq data also depend on the complexity of clinical endpoints. For example, endpoints C_SEX_All (sex of patients) and D_FAV_All (unfavorable and favorable patients for extreme disease course) of the SEQC NB data set and SEX and Cyto genetic risk of the TCGA AML Data set were the easiest to predict and showed the highest transferability; while E_EFS_HR (event-free survival for high-risk patients) and F_OS_HR (overall survival for high-risk patients) of the SEQC NB data set and EFS and OS of the TCGA AML data set were the most difficult to predict and exhibited the lowest transferability. Even though the complexity of cross-platform gene mappings can cause large variation in absolute gene-expression values between microarrays and RNA-Seq, it had minimal effect on the observed transferability of signature genes. In addition, the prediction performance of models developed using three distinct gene mappings was very similar to each other, indicating that there was a lot of redundant information in both microarray and RNA-Seq data and that only a fraction of all the available data is necessary to derive models with good cross-platform predictions.

## Conclusions

The analyses demonstrated that microarray models can directly predict RNA-Seq-profiled samples if the gene-expression data were z-score preprocessed before modeling and prediction and that the signature genes of a model developed from one platform can be directly transferred to the other platform for model development. However, it is difficult to directly use the models developed from RNA-Seq data to predict microarray-profiled samples. Our study offers a viable option for the proper use of legacy microarray data, microarray-based predictive models and biomarkers in the RNA-Seq era and demonstrated a means to utilize RNA-Seq-based signature genes in microarray data analysis.

## Methods and materials

### Neuroblastoma (NB) data set from the FDA SEquencing Quality Control (SEQC) project

The FDA SEQC NB data set contains 498 NB samples that were independently profiled with Agilent customized 4 × 44 K oligonucleotide microarrays and Illumina HiSeq 2000 RNA-Seq. In the FDA SEQC NB study, six binary clinically relevant endpoints and four continuous survival times were defined among the 498 NB samples (Table 1). For each clinical endpoint, samples were assigned to either a training set or a validation set, with varying numbers of positive and negative samples in each set (Table 1). Detailed information on the NB samples, clinical parameters, microarrays and RNA-Seq assays were described elsewhere. The data set can be obtained from GEO database with series accession numbers GSE49710 and GSE62564 for microarray and RNA-Seq data, respectively.

### Acute myeloid leukemia (AML) data from The Cancer Genome Atlas (TCGA)

The TCGA AML [17] data set includes 175 paired Affymetrix HG-U133_plus_2 microarrays and Illumina RNA-Seq assays after cleaning up the cytogenetic risk endpoint. The microarray and RNA-Seq data were generated from the same set of AML tumor RNA samples. The microarray MAS5 normalized data and RNA-Seq RPKM data were downloaded from [31,32], respectively. The clinical information of patients was downloaded from [31].

We used patients’ sex and cytogenetic risk as two binary endpoints for binary endpoint prediction analysis and used event-free survival (EFS) and overall survival (OS) times as two continuous responses with Cox proportional hazards regression to predict patients’ potential survival risk based on gene expression data. The training and validation sets were constructed as follow: (1) since the original cytogenetic risk includes good, intermediate, and poor three levels, we combined the intermediate and poor levels together to form a new ‘poor’ level and used with the original good level for binary endpoint prediction analysis; (2) Then randomly split the patients in the two cytogenetic risk groups into a training set (17 good +51 intermediate +21 poor) and a validation set (16 good +50 intermediate +20 poor). The same splitting was also used for the endpoint sex. The training set includes 43 female and 46 male patients, while the validation set contains 39 female and 47 male patients.

### NCTR rat toxicogenomics data set

The NCTR rat toxicogenomics data set includes eight microarray and eight RNA-Seq assays. The microarray and RNA-Seq data were generated from exactly the same set of RNA samples isolated from the kidneys of four aristolochic acid-treated and four control rats [20]. The microarray assays were done in the MicroArray Quality Control phase I (MAQC-I) validation study [21] with Affymetrix Rat_230_2 arrays and the RNA-Seq data were generated in another study [20] with the Illumina GA II platform and single-end 36 base pairs length protocol. The microarray data were previously processed using MAS5 [21]. The RNA-Seq reads were aligned against UCSC Rat genome rn4 [33] using Novoalign v2.08.1 [34] and gene counts were quantified and normalized with the P2 pipeline [22]. The microarray and RNA-Seq data can be downloaded from GEO database with series accession numbers GSE5350 and GSE21210, respectively.

### FDA SEquencing Quality Control (SEQC) main study data and MicroArray Quality Control phase I (MAQC-I) main study data

Microarray data came from the FDA MAQC main study [23] and consisted of data from Affymetrix HG-U133_Plus_2 microarrays. The RNA-Seq data were from the FDA SEQC main study [16] using the Illumina HiSeq 2000 platform. The microarray data were generated by Affymetrix site 1 in the MAQC study, while the RNA-Seq data were generated by site BGI in the SEQC study. Both sets of data were generated from the same set of four human RNA samples, that is, Universal Human Reference RNA (UHRR, Agilent), Human Brain Reference RNA (HBRR, Life Technologies), and mixtures C and D of UHRR and HBRR samples in a ratio of 3:1 and 1:3, respectively. The HG-U133_Plus_2 arrays were normalized with MAS5 algorithm. The RNA-Seq data were generated with Illumina HiSeq 2000 using the paired-end 100 bp TruSeq v3 RNA-Seq protocol and were analyzed with the P2 pipeline [22] using UCSC human genome hg19 as reference. Gene counts were normalized into reads per million (RPM) with a global scaling approach [35]. The microarray and RNA-Seq data can be obtained from GEO database with series accession numbers GSE5350 and GSE47774, respectively.

### FDA SEQC rat toxicogenomics data

The SEQC rat toxicogenomics data contains 62 rat liver RNA samples. Each individual RNA sample was separately assayed with Affymetrix Rat_230_2 arrays and Illumina HiScanSQ/HiSeq 2000 RNA-Seq. The microarray data were generated and normalized in the National Toxicology Program DrugMatrix Database. Details about the data generation and normalization can be found elsewhere [22]. Here, we directly downloaded MAS5 normalized data from the DrugMatrix ftp site [36]. For RNA-Seq analysis, the paired-end 100 base pair Illumina TruSeq RNA-Seq protocol was used. The RNA-Seq reads were analyzed with six different bioinformatics pipelines with different references used by six independent data analysis teams (Table 4), that is, P1 (NCBI magic), P2 (Novoalign with RefSeq gene models), P3 (Bwa + RefSeq RNAs), P4 (Tophat + HTSeq with RefSeq gene models), P5 (Bowtie + RSEM with Ensembl gene models), and P6 (Tophat + cufflinks *de novo* assembly). Details about RNA-Seq reads generation, alignment, and gene counting can be found elsewhere [22]. The microarray and RNA-Seq data can be downloaded from GEO database with series accession number GSE47875 and GSE55347, respectively.

### Cross-platform gene mapping between microarray and RNA-Seq

The method used for cross-platform gene mapping between microarray probes/probe sets and RNA-Seq genes is depicted in Figure 3a. The workflow was implemented in a software tool that can be obtained upon request. Two mapping methods, public gene ID mapping and genome location mapping, were implemented in the software tool. To use both approaches, the array probes/probe sets annotation information for individual microarray is required. For Affymetrix arrays, annotation files, usually in CSV format, are available at the Affymetrix web site [37]. Information for RNA-Seq genes can be in one of two formats: (1) a gene ID list file containing one of the following public gene IDs: RefSeq transcript ID, gene symbol, Ensembl gene ID, or Entrez gene ID; or (2) a GTF/GFF file generated by RNA-Seq pipelines during *de novo* assembly or used by RNA-Seq pipelines for quantification of gene expression.

To map by one of the four types of public gene IDs, each array probe/probe set was examined by comparing the gene ID or ID list in the corresponding array type annotation file to all RNA-Seq genes. In order to map with a genome location mapping approach, the coordinates in the ‘Alignments’ column of array type annotation files were used to calculate the overlap between each microarray probe set and all exons of each RNA-Seq gene. A microarray probe set was considered to be mapped to a RNA-Seq gene if the length of the overlap between the coordinates specified in the annotation file and an exon of an RNA-Seq gene was greater than 40 base pairs, or if it overlapped with at least two exons of the RNA-Seq gene. After mapping, both microarray probes/probe sets and RNA-Seq genes were separately classified into four different groups: A, B, C, and D (Table 2).

### Microarray gene annotation files

The Agilent customized 4 × 44 K oligonucleotide microarray annotation file was obtained from the GEO database with series accession number GSE49710.

The microarray probe set annotation files for Rat230_2 and HG-U133_Plus_2 were downloaded from the Affymetrix web site [37]. Both files were created on 9 June 2011 by using the Netaffx™ Annotation software. The HG-U133_Plus_2 and Rat230_2 array types were annotated with human genome UCSC version hg19 (or NCBI GRCh37) and rat genome UCSC version rn4, respectively. Other reference databases used for both array types for annotation included Ensembl version 60, GenBank version 180, and RefSeq release 41.

### T-index for assessing model transferability

We used the T-index [24] score to measure the transferability of predictive models and signature genes. The T-index score has a value between 0 and 1. A larger T-index score means better transferability across platforms and a T-index score less than 0.5 indicates that the transferability is due to chance. The T-index sxcore was calculated according to formula (1):

1 $$ {T}^A=\frac{1}{N}{\displaystyle \sum_{k=1}^N{P}_k^A}\left[1-\frac{\frac{1}{N}{\displaystyle \sum_{k=1}^N\left({P}_k^A-{P}_k^B\right)}}{e^{-s.d.}}\right] $$

where *T*^*A*^ is a metric for estimating the transferability of the models developed from platform *A* to the models for platform *B. N* is the total number of models (500 in this study). ${P}_k^A$ and ${P}_k^B$ are prediction accuracies of the models developed from platform *A* and *B*, respectively. s.d. is the standard deviation of (${P}_k^A-{P}_k^B$).

### RNA-Seq pipelines and gene models

Table 4 lists the RNA-Seq pipelines and gene models used for each RNA-Seq data set used in the study.

### Data availability

The SEQC NB microarray gene expression data and RNA-Seq log_2_RPM used in this study can be downloaded from the GEO database with series accession number GSE49710 and GSE62564, respectively. The TCGA AML clinical information, microarray and RNA-Seq data can be separately downloaded using links https://tcga-data.nci.nih.gov/docs/publications/laml_2012/clinical_patient_laml.tsv, https://tcga-data.nci.nih.gov/docs/publications/laml_2012/HG-U133_Plus_2.Level_2.tgz and https://tcga-data.nci.nih.gov/docs/publications/laml_2012/laml.rnaseq.179_v1.0_gaf2.0_rpkm_matrix.txt.tcgaID.txt.gz. The NCTR rat toxicogenomics microarray and RNA-Seq data can be obtained with GEO series accession numbers GSE5350 and GSE21210, respectively. The MAQC-I main study and the SEQC main study data can be downloaded from GEO database with series accession numbers GSE5350 and GSE47774, respectively. The SEQC rat toxicogenomics microarray and RNA-Seq data have been deposited in GEO database under series accession numbers GSE47875 and GSE55347, respectively.

## Additional files

Additional file 1: Figure S1. Comparison of the number of samples profiled by expression microarray and RNA-Seq in the Gene Expression Omnibus (GEO) database. The numbers of samples for expression profiling by microarray or by high throughput sequencing (RNA-Seq) were collected from the GEO database on 28 April 2014. In the GEO database, the start dates for expression microarray and RNA-Seq data accumulation are 2001 and 2006, respectively. Each bar (blue or red) represents the number of samples for expression profiling cumulated in the GEO database since the start date (2001 and 2006 for microarray and RNA-Seq, respectively). The dashed blue and red lines are the trend lines fitted with the ‘Polynomial’ and ‘Power’ options, respectively, in Excel. The bars after 2013 are the projections of the trend lines fitted with current the GEO data. Additional file 2: Figure S2. The consistency of Affymetrix microarray and RNA-Seq gene expression levels for MAQC reference RNA samples. The intensities of Affymetrix microarray probe sets in three mapping groups A, B, and C are separately compared to the corresponding RNA-Seq gene counts in panels **(a)**, **(b)**, and **(c)** for one of the four MicroArray Quality Control (MAQC) human RNA samples. The mappings from microarray probe sets to RNA-Seq genes are based on the genome location mapping approach. The microarray data are from MAQC-I Affymetrix HG-U133_Plus_2 arrays with MAS5-normalized probe set intensities, and the RNA-Seq reads are from the FDA SEquencing Quality Control (SEQC) Illumina HiSeq 2000 with gene counts from the P2 pipeline (Novoalign with RefSeq human gene models). Additional file 3: Figure S3. The consistency of Affymetrix HG-U133_Plus_2 microarray and RNA-Seq gene expression levels for the acute myeloid leukemia (AML) RNA samples. The intensities of Affymetrix array probe sets in three mapping groups A, B, and C are separately compared to the corresponding RNA-Seq gene counts in panels **(a)**, **(b)**, and **(c)** for one of the 175 acute myeloid leukemia (AML) RNA samples from The Cancer Genome Atlas (TCGA) acute myeloid leukemia study. The microarray data are MAS5 normalized and the RNA-Seq data are scaled as RPKM. The mappings from array probe sets to RNA-Seq genes are based on the gene ID mapping approach. Additional file 4: Figure S4. The consistency of Agilent microarray and RNA-Seq gene expression levels for human RNA samples. The intensities of Agilent array probes in three mapping groups A, B, and C are separately compared to the corresponding RNA-Seq gene counts in panels **(a)**, **(b)**, and **(c)** for one of the 498 neuroblastoma RNA samples from the FDA SEquencing Quality Control (SEQC) project. The mappings from Agilent probes to RNA-Seq genes are based on the gene ID mapping approach. The microarray data are from Agilent customized 4 × 44 K oligonucleotide arrays, and RNA-Seq reads are from Illumina HiSeq 2000 with gene counts from the P2 pipeline (Novoalign with RefSeq human gene models). Additional file 5: Figure S5. The consistency of microarray gene expression levels and six sets of RNA-Seq gene counts derived from the same set of RNA-Seq raw data but using a diversity of RNA-Seq data analysis approaches. The MAS5 normalized microarray gene expression levels of mapping groups A, B, and C are plotted against the corresponding RNA-Seq measurements generated by six independent data analysis teams with a variety of bioinformatics pipelines and references, that is, **(a)** P1 (NCBI Magic), **(b)** P2 (Novoalign with RefSeq rat gene models), **(c)** P3 (BWA + RefSeq Rat RNAs), **(d)** P4 (Tophat + HTSeq with RefSeq rat gene models), **(e)** P5 (Bowtie + RSEM with Ensembl rat gene models), and **(f)** P6 (Tophat + Cufflinks *de novo* assembly). The mappings from microarrays to P1, P2, P3, and P4 gene sets are based on the gene ID mapping approach, while to P5 and P6 gene sets on the genome location mapping. The data set containing 62 rat liver RNAs is from sample profiling in the FDA SEquencing Quality Control toxicogenomics study with separate assays for each individual RNA sample from Affymetrix Rat_230_2 arrays and Illumina HiScanSQ/HiSeq 2000 RNA-Seq. In each of the six subpanels, gene expression measurements for mapping groups A, B, and C from microarrays are plotted against those from RNA-Seq in scatter plots (1), (2), and (3), respectively. Additional file 6: Figure S6. A performance comparison of nearest shrunken centroids (NSC) models and their corresponding transferred models based on the SEQC NB data. For each of the six binary clinical endpoints and each of the three mapping groups A, B, and C, a set of 500 NSC models were developed from microarray training data and used to predict microarray validation samples. The signature genes of each of the 500 microarray models were then used with all RNA-Seq training data for those genes to build an untrained RNA-Seq model to predict RNA-Seq validation samples. Finally, the average prediction accuracies of the 500 microarray models are plotted against those of the 500 corresponding RNA-Seq models **(a)**, with the per sample agreement better than chance evaluated with the Kappa statistic as shown in **(b)**. The transferability of the signature genes from RNA-Seq back to microarray data was conversely calculated. The 500 NSC models trained from RNA-Seq data were used to predict RNA-Seq validation samples. Then the signature genes of each RNA-Seq model were used with all microarray training data for those genes to build an untrained NSC model to predict microarray validation samples. The average accuracies of the 500 RNA-Seq models were then compared to those of the 500 corresponding microarray models **(c)**, with the per sample agreement better than chance assessed with the Kappa statistic as shown in **(d)**. The six symbols in each panel represent the six binary clinical endpoints with green, blue, and orange colors denoting mapping groups A, B, and C, respectively. In panels **(b)** and **(d)**, each symbol denotes the average Kappa statistic of the 500 pairs of model predictions; and each error bar shows the 95% confidence interval (CI) for the mean Kappa statistic. Each CI was calculated with the bootstrap estimation. No significant difference is observed between trained microarrays models and transferred RNA-Seq models (paired t-test *P* is 0.841) and between the trained RNA-Seq models and the transferred microarray models (paired t-test *P* is 0.058). Additional file 7: Figure S7. A performance comparison of support vector machine (SVM) models and their corresponding transferred models based on the SEQC NB data. For each of the six binary clinical endpoints and each of the three mapping groups A, B, and C, a set of 500 SVM models were developed from microarray training data and used to predict microarray validation samples. The signature genes of each of the 500 models were then used with all RNA-Seq training data for those genes to build a RNA-Seq model to predict RNA-Seq validation samples. Finally, the average prediction accuracies of the 500 microarray models are plotted against those of the 500 corresponding RNA-Seq models **(a)**, with the per sample agreement better than chance evaluated with the Kappa statistic as shown in **(b)**. The transferability of the signature genes from RNA-Seq back to microarray data was conversely computed. The 500 SVM models trained from RNA-Seq data were used to predict RNA-Seq validation samples. Then the signature genes of each RNA-Seq model were used with all microarray training data for those genes to build a microarray model to predict microarray validation samples. The average accuracies of the 500 RNA-Seq models were then compared to those of the 500 corresponding microarray models **(c)**, with the per sample agreement better than chance assessed with the Kappa statistic as shown in **(d)**. The six symbols in each panel represent the six binary clinical endpoints with green, blue, and orange colors denoting mapping groups A, B, and C, respectively. In panels **(b)** and **(d)**, each symbol denotes the average Kappa statistic of the 500 pairs of models; and each error bar shows the 95% confidence interval (CI) for the mean Kappa statistic. Each CI was calculated with the bootstrap estimation. No significant difference is observed between trained microarray models and transferred untrained RNA-Seq models (paired t-test *P* is 0.557) and between trained RNA-Seq models and transferred untrained microarray models (paired t-test *P* is 0.158). Additional file 8: Table S1. Performance metrics for the assessment of cross-platform transferability of signature genes of NSC models based on the SEQC NB data. Additional file 9: Table S2. Performance metrics for the assessment of cross-platform transferability of signature genes of SVM models based on the SEQC NB data. Additional file 10: Figure S8. A performance comparison of nearest shrunken centroids (NSC) models in predicting microarray and RNA-Seq validation data based on the SEQC NB data. In the comparison, both microarray log2 intensity and RNA-Seq log2 count were per sample z-scored. For each of the six binary clinical endpoints and each of the two mapping groups A and B, a set of 500 NSC models were developed from microarray and RNA-Seq training data independently. Each set of NCS models were then used to predict both microarray and RNA-Seq validation samples. The average prediction accuracies of the 500 microarray-based models in prediction microarray data were plotted against those in predicting RNA-Seq data **(a)**, with per sample agreement better than chance assessed with the Kappa statistic as shown in **(b)**; while the average accuracies of the 500 RNA-Seq-based models in predicting RNA-Seq data were compared to those in predicting microarray data **(c)**, with per sample agreement better than chance evaluated with the Kappa statistic as shown in **(d)**. The six symbols in each panel represent the six binary clinical endpoints with green and blue colors denoting mapping groups A and B, respectively. In panels **(b)** and **(d)**, each symbol denotes the average Kappa statistic of 500 pairs of prediction results; and each error bar shows the 95% confidence interval (CI) for the mean Kappa statistic. Each CI was calculated with the bootstrap estimation. Additional file 11: Table S3. The performance of NSC models in predicting microarray and RNA-Seq validation samples based on the SEQC NB data. Additional file 12: Figure S9. A performance comparison of support vector machine (SVM) models in predicting microarray and RNA-Seq validation data based on the SEQC NB data. In the comparison, both microarray log2 intensity and RNA-Seq log2 count were per sample z-score transformed. For each of the six binary clinical endpoints and each of the two mapping groups A and B, a set of 500 SVM models were developed from microarray and RNA-Seq training data independently. Each set of models were then used to predict both microarray and RNA-Seq validation samples. The average prediction accuracies of the 500 microarray-based models in prediction microarray data were plotted against those in predicting RNA-Seq data **(a)**, with per sample agreement better than chance assessed with the Kappa statistic as shown in **(b)**; while the average accuracies of the 500 RNA-Seq-based models in predicting RNA-Seq data were compared to those in predicting microarray data **(c)**, with per sample agreement evaluated with the Kappa statistic as shown in **(d)**. The six symbols in each panel represent the six binary clinical endpoints with green and blue colors denoting mapping groups A and B, respectively. In panels **(b)** and **(d)**, each symbol denotes the average Kappa statistic of 500 pairs of prediction results; and each error bar shows the 95% confidence interval (CI) for the mean Kappa statistic. Each CI was calculated with the bootstrap estimation. Additional file 13: Table S4. The performance of SVM models in predicting microarray and RNA-Seq validation samples based on the SEQC NB data. Additional file 14: Figure S10. A performance comparison of k-nearest neighbors (k-NN) models in predicting microarray and RNA-Seq validation samples, based on the SEQC NB Data without per sample z-score transformation. In the comparison, microarray and RNA-Seq data were log2 intensity data and log2 counts, respectively. For each of the six binary clinical endpoints and each of the two mapping groups A and B, a set of 500 k-NN models were developed from microarray and RNA-Seq training data independently. Each set of k-NNs models were then used to predict both microarray and RNA-Seq validation samples. The average prediction accuracies of the 500 microarray-based models in prediction microarray data were plotted against those in predicting RNA-Seq data **(a)**, with per sample agreement better than chance assessed with the Kappa statistic as shown in **(b)**; while the average accuracies of the 500 RNA-Seq-based models in predicting RNA-Seq data were compared to those in predicting microarray data **(c)**, with per sample agreement better than chance evaluated with the Kappa statistic as shown in **(d)**. The six symbols in each panel represent the six binary clinical endpoints with green and blue colors denoting mapping groups A and B, respectively. In panels **(b)** and **(d)**, each symbol denotes the average Kappa statistic of 500 pairs of prediction results; and each error bar shows the 95% confidence interval (CI) for the mean Kappa statistic. Each CI was calculated with the bootstrap estimation. Additional file 15: Figure S11. A performance comparison of nearest shrunken centroids (NSC) models in predicting microarray and RNA-Seq validation samples, based on the SEQC NB Data without per sample z-score transformation. In the comparison, microarray and RNA-Seq data were log2 intensity data and log2 counts, respectively. For each of the six binary clinical endpoints and each of the two mapping groups A and B, a set of 500 NSC models were developed from microarray and RNA-Seq training data independently. Each set of NCS models were then used to predict both microarray and RNA-Seq validation samples. The average prediction accuracies of the 500 microarray-based models in prediction microarray data were plotted against those in predicting RNA-Seq data **(a)**, with per sample agreement better than chance assessed with the Kappa statistic as shown in **(b)**; while the average accuracies of the 500 RNA-Seq-based models in predicting RNA-Seq data were compared to those in predicting microarray data **(c)**, with per sample agreement better than chance evaluated with the Kappa statistic as shown in **(d)**. The six symbols in each panel represent the six binary clinical endpoints with green and blue colors denoting mapping groups A and B, respectively. In panels **(b)** and **(d)**, each symbol denotes the average Kappa statistic of 500 pairs of prediction results; and each error bar shows the 95% confidence interval (CI) for the mean Kappa statistic. Each CI was calculated with the bootstrap estimation. Additional file 16: Figure S12. A performance comparison of support vector machine (SVM) models in predicting microarray and RNA-Seq validation samples, based on the SEQC NB Data without per sample z-score transformation. In the comparison, microarray and RNA-Seq data were log2 intensity data and log2 counts, respectively. For each of the six binary clinical endpoints and each of the two mapping groups A and B, a set of 500 SVM models were developed from microarray and RNA-Seq training data independently. Each set of models were then used to predict both microarray and RNA-Seq validation samples. The average prediction accuracies of the 500 microarray-based models in prediction microarray data were plotted against those in predicting RNA-Seq data **(a)**, with per sample agreement better than chance assessed with the Kappa statistic as shown in **(b)**; while the average accuracies of the 500 RNA-Seq-based models in predicting RNA-Seq data were compared to those in predicting microarray data **(c)**, with per sample agreement better than chance evaluated with the Kappa statistic as shown in **(d)**. The six symbols in each panel represent the six binary clinical endpoints with green and blue colors denoting mapping groups A and B, respectively. In panels **(b)** and **(d)**, each symbol denotes the average Kappa statistic of 500 pairs of prediction results; and each error bar shows the 95% confidence interval (CI) for the mean Kappa statistic. Each CI was calculated with the bootstrap estimation. Additional file 17: Table S5. Concordance indices and P values for the assessment of cross-platform transferability of signature genes of Cox models based on the SEQC NB data. Additional file 18: Table S6. The performance of Cox proportional hazards models developed from one-platform in predicting microarray and RNA-Seq validation samples based on the SEQC NB data. Additional file 19: Figure S13. A performance comparison of nearest shrunken centroids (NSC) models and their corresponding transferred models based on the TCGA AML data. For each of the two binary clinical endpoints and each of the three mapping groups A, B, and C, a set of 500 NSC models were developed from microarray training data and used to predict microarray validation samples. The signature genes of each of the 500 microarray models were then used with all RNA-Seq training data for those genes to build an untrained RNA-Seq model to predict RNA-Seq validation samples. Finally, the average prediction accuracies of the 500 microarray models are plotted against those of the 500 corresponding RNA-Seq models **(a)**, with the per sample agreement better than chance evaluated with the Kappa statistic as shown in **(b)**. The transferability of the signature genes from RNA-Seq back to microarray data was conversely calculated. The 500 NSC models trained from RNA-Seq data were used to predict RNA-Seq validation samples. Then the signature genes of each RNA-Seq model were used with all microarray training data for those genes to build an untrained NSC model to predict microarray validation samples. The average accuracies of the 500 RNA-Seq models were then compared to those of the 500 corresponding microarray models **(c)**, with the per sample agreement better than chance assessed with the Kappa statistic as shown in **(d)**. The two symbols in each panel represent the two binary clinical endpoints with green, blue, and orange colors denoting mapping groups A, B, and C, respectively. In panels **(b)** and **(d)**, each symbol denotes the average Kappa statistic of the 500 pairs of model predictions; and each error bar shows the 95% confidence interval (CI) for the mean Kappa statistic. Each CI was calculated with the bootstrap estimation. No significant difference is observed between trained microarrays models and transferred RNA-Seq models (paired t-test *P* is 0.354) and between the trained RNA-Seq models and the transferred microarray models (paired t-test *P* is 0.106). Additional file 20: Figure S14. A performance comparison of support vector machine (SVM) models and their corresponding transferred models based on the TCGA AML data. For each of the two binary clinical endpoints and each of the three mapping groups A, B, and C, a set of 500 SVM models were developed from microarray training data and used to predict microarray validation samples. The signature genes of each of the 500 models were then used with all RNA-Seq training data for those genes to build a RNA-Seq model to predict RNA-Seq validation samples. Finally, the average prediction accuracies of the 500 microarray models are plotted against those of the 500 corresponding RNA-Seq models **(a)**, with the per sample agreement better than chance evaluated with the Kappa statistic as shown in **(b)**. The transferability of the signature genes from RNA-Seq back to microarray data was conversely computed. The 500 SVM models trained from RNA-Seq data were used to predict RNA-Seq validation samples. Then the signature genes of each RNA-Seq model were used with all microarray training data for those genes to build a microarray model to predict microarray validation samples. The average accuracies of the 500 RNA-Seq models were then compared to those of the 500 corresponding microarray models **(c)**, with the per sample agreement better than chance assessed with the Kappa statistic as shown in **(d)**. The two symbols in each panel represent the two binary clinical endpoints with green, blue, and orange colors denoting mapping groups A, B, and C, respectively. In panels (b) and (d), each symbol denotes the average Kappa statistic of the 500 pairs of models; and each error bar shows the 95% confidence interval (CI) for the mean Kappa statistic. Each CI was calculated with the bootstrap estimation. No significant difference is observed between trained microarray models and transferred untrained RNA-Seq models (paired t-test *P* is 0.305) and between trained RNA-Seq models and transferred untrained microarray models (paired t-test *P* is 0.022). Additional file 21: Table S7. Concordance indices and p values for the assessment of cross-platform transferability of signature genes of Cox models based on the TCGA AML data. Additional file 22: Figure S15. A performance comparison of nearest shrunken centroids (NSC) models in predicting microarray and RNA-Seq validation data based on the TCGA AML data. In the comparison, both microarray log2 intensity and RNA-Seq log2 count were per sample z-scored. For each of the two clinical binary endpoints and each of the two mapping groups A and B, a set of 500 NSC models were developed from microarray and RNA-Seq training data independently. Each set of NCS models were then used to predict both microarray and RNA-Seq validation samples. The average prediction accuracies of the 500 microarray-based models in prediction microarray data were plotted against those in predicting RNA-Seq data **(a)**, with per sample agreement better than chance assessed with the Kappa statistic as shown in **(b)**; while the average accuracies of the 500 RNA-Seq-based models in predicting RNA-Seq data were compared to those in predicting microarray data **(c)**, with per sample agreement better than chance evaluated with the Kappa statistic as shown in **(d)**. The two symbols in each panel represent the two binary clinical endpoints with green and blue colors denoting mapping groups A and B, respectively. In panels **(b)** and **(d)**, each symbol denotes the average Kappa statistic of 500 pairs of prediction results; and each error bar shows the 95% confidence interval (CI) for the mean Kappa statistic. Each CI was calculated with the bootstrap estimation. Additional file 23: Figure S16. A performance comparison of support vector machine (SVM) models in predicting microarray and RNA-Seq validation data based on the TCGA AML data. In the comparison, both microarray log2 intensity and RNA-Seq log2 count were per sample z-score transformed. For each of the two binary clinical endpoints and each of the two mapping groups A and B, a set of 500 SVM models were developed from microarray and RNA-Seq training data independently. Each set of models were then used to predict both microarray and RNA-Seq validation samples. The average prediction accuracies of the 500 microarray-based models in prediction microarray data were plotted against those in predicting RNA-Seq data **(a)**, with per sample agreement better than chance assessed with the Kappa statistic as shown in **(b)**; while the average accuracies of the 500 RNA-Seq-based models in predicting RNA-Seq data were compared to those in predicting microarray data **(c)**, with per sample agreement evaluated with the Kappa statistic as shown in **(d)**. The two symbols in each panel represent the two binary clinical endpoints with green and blue colors denoting mapping groups A and B, respectively. In panels **(b)** and **(d)**, each symbol denotes the average Kappa statistic of 500 pairs of prediction results; and each error bar shows the 95% confidence interval (CI) for the mean Kappa statistic. Each CI was calculated with the bootstrap estimation. Additional file 24: Figure S17. A performance comparison of k-nearest neighbors (k-NN) models in predicting microarray and RNA-Seq validation samples, based on the TCGA AML data without per sample z-score transformation. In the comparison, microarray and RNA-Seq data were log2 intensity data and log2 counts, respectively. For each of the two binary clinical endpoints and each of the two mapping groups A and B, a set of 500 k-NN models were developed from microarray and RNA-Seq training data independently. Each set of k-NNs models were then used to predict both microarray and RNA-Seq validation samples. The average prediction accuracies of the 500 microarray-based models in prediction microarray data were plotted against those in predicting RNA-Seq data **(a)**, with per sample agreement better than chance assessed with the Kappa statistic as shown in **(b)**; while the average accuracies of the 500 RNA-Seq-based models in predicting RNA-Seq data were compared to those in predicting microarray data **(c)**, with per sample agreement better than chance evaluated with the Kappa statistic as shown in **(d)**. The two symbols in each panel represent the two binary clinical endpoints with green and blue colors denoting mapping groups A and B, respectively. In panels **(b)** and **(d)**, each symbol denotes the average Kappa statistic of 500 pairs of prediction results; and each error bar shows the 95% confidence interval (CI) for the mean Kappa statistic. Each CI was calculated with the bootstrap estimation. Additional file 25: Figure S18. A performance comparison of nearest shrunken centroids (NSC) models in predicting microarray and RNA-Seq validation samples, based on the TCGA AML data without per sample z-score transformation. In the comparison, microarray and RNA-Seq data were log2 intensity data and log2 counts, respectively. For each of the two binary clinical endpoints and each of the two mapping groups A and B, a set of 500 NSC models were developed from microarray and RNA-Seq training data independently. Each set of NCS models were then used to predict both microarray and RNA-Seq validation samples. The average prediction accuracies of the 500 microarray-based models in prediction microarray data were plotted against those in predicting RNA-Seq data **(a)**, with per sample agreement better than chance assessed with the Kappa statistic as shown in **(b)**; while the average accuracies of the 500 RNA-Seq-based models in predicting RNA-Seq data were compared to those in predicting microarray data **(c)**, with per sample agreement better than chance evaluated with the Kappa statistic as shown in **(d)**. The two symbols in each panel represent the two binary clinical endpoints with green and blue colors denoting mapping groups A and B, respectively. In panels **(b)** and **(d)**, each symbol denotes the average Kappa statistic of 500 pairs of prediction results; and each error bar shows the 95% confidence interval (CI) for the mean Kappa statistic. Each CI was calculated with the bootstrap estimation. Additional file 26: Figure S19. A performance comparison of support vector machine (SVM) models in predicting microarray and RNA-Seq validation samples, based on the TCGA AML data without per sample z-score transformation. In the comparison, microarray and RNA-Seq data were log2 intensity data and log2 counts, respectively. For each of the two binary clinical endpoints and each of the two mapping groups A and B, a set of 500 SVM models were developed from microarray and RNA-Seq training data independently. Each set of models were then used to predict both microarray and RNA-Seq validation samples. The average prediction accuracies of the 500 microarray-based models in prediction microarray data were plotted against those in predicting RNA-Seq data **(a)**, with per sample agreement better than chance assessed with the Kappa statistic as shown in **(b)**; while the average accuracies of the 500 RNA-Seq-based models in predicting RNA-Seq data were compared to those in predicting microarray data **(c)**, with per sample agreement better than chance evaluated with the Kappa statistic as shown in **(d)**. The two symbols in each panel represent the two binary clinical endpoints with green and blue colors denoting mapping groups A and B, respectively. In panels **(b)** and **(d)**, each symbol denotes the average Kappa statistic of 500 pairs of prediction results; and each error bar shows the 95% confidence interval (CI) for the mean Kappa statistic. Each CI was calculated with the bootstrap estimation. Additional file 27: Table S8. The performance of Cox proportional hazards models developed from one-platform in predicting microarray and RNA-Seq validation samples based on the TCGA AML data.

## Abbreviations

AML: Acute myeloid leukemia
k-NN: k-nearest neighbors
MAQC: MicroArray quality control
NB: Neuroblastoma
NSC: Nearest shrunken centroids
SEQC: SEquencing quality control
SVM: Support vector machine
TCGA: The cancer genome atlas
