# Supplementary material for: An investigation of biomarkers derived from legacy microarray data for their utility in the RNA-seq era
Source: Genome Biol. 2014 Dec 3;15(12):3273. doi: 10.1186/s13059-014-0523-y (PMC4290828; doi:10.1186/s13059-014-0523-y)
Supplement: Additional file 1: Figure S1. — Comparison of the number of samples profiled by expression microarray and RNA-Seq in the Gene Expression Omnibus (GEO) database. The numbers of samples for expression profiling by microarray or by high throughput sequencing (RNA-Seq) were collected from the GEO database on 28 April 2014. In the GEO database, the start dates for expression microarray and RNA-Seq data accumulation are 2001 and 2006, respectively. Each bar (blue or red) represents the number of samples for expression profiling cumulated in the GEO database since the start date (2001 and 2006 for microarray and RNA-Seq, respectively). The dashed blue and red lines are the trend lines fitted with the ‘Polynomial’ and ‘Power’ options, respectively, in Excel. The bars after 2013 are the projections of the trend lines fitted with current the GEO data. [file 13059_2014_523_MOESM1_ESM.doc]

## Figure S1. Comparison of the number of samples profiled by expression microarray and RNA-Seq in the Gene Expression Omnibus (GEO) database.

The numbers of samples for expression profiling by microarray or by high throughput sequencing (RNA-Seq) were collected from the GEO database on 28 April 2014. In the GEO database, the start dates for expression microarray and RNA-Seq data accumulation are 2001 and 2006, respectively. Each bar (blue or red) represents the number of samples for expression profiling cumulated in the GEO database since the start date (2001 and 2006 for microarray and RNA-Seq, respectively). The dashed blue and red lines are the trend lines fitted with the ‘Polynomial’ and ‘Power’ options respectively in Excel. The bars after 2013 are the projections of the trend lines fitted with current the GEO data.
