# Supplementary material for: An investigation of biomarkers derived from legacy microarray data for their utility in the RNA-seq era
Source: Genome Biol. 2014 Dec 3;15(12):3273. doi: 10.1186/s13059-014-0523-y (PMC4290828; doi:10.1186/s13059-014-0523-y)
Supplement: Additional file 2: Figure S2. — The consistency of Affymetrix microarray and RNA-Seq gene expression levels for MAQC reference RNA samples. The intensities of Affymetrix microarray probe sets in three mapping groups A, B, and C are separately compared to the corresponding RNA-Seq gene counts in panels (a), (b), and (c) for one of the four MicroArray Quality Control (MAQC) human RNA samples. The mappings from microarray probe sets to RNA-Seq genes are based on the genome location mapping approach. The microarray data are from MAQC-I Affymetrix HG-U133_Plus_2 arrays with MAS5-normalized probe set intensities, and the RNA-Seq reads are from the FDA SEquencing Quality Control (SEQC) Illumina HiSeq 2000 with gene counts from the P2 pipeline (Novoalign with RefSeq human gene models). [file 13059_2014_523_MOESM2_ESM.doc]

## Figure S2. The consistency of Affymetrix microarray and RNA-Seq gene expression levels for MAQC reference RNA samples.

The intensities of Affymetrix microarray probe sets in three mapping groups A, B, and C are separately compared to the corresponding RNA-Seq gene counts in panels **(a)**, **(b)**, and **(c)** for one of the four MicroArray Quality Control (MAQC) human RNA samples. The mappings from microarray probe sets to RNA-Seq genes are based on the genome location mapping approach. The microarray data are from MAQC-I Affymetrix HG-U133_Plus_2 arrays with MAS5-normalized probe set intensities, and the RNA-Seq reads are from the FDA SEquencing Quality Control (SEQC) Illumina HiSeq 2000 with gene counts from the P2 pipeline (Novoalign with RefSeq human gene models).
