# Supplementary material for: An investigation of biomarkers derived from legacy microarray data for their utility in the RNA-seq era
Source: Genome Biol. 2014 Dec 3;15(12):3273. doi: 10.1186/s13059-014-0523-y (PMC4290828; doi:10.1186/s13059-014-0523-y)
Supplement: Additional file 3: Figure S3. — The consistency of Affymetrix HG-U133_Plus_2 microarray and RNA-Seq gene expression levels for the acute myeloid leukemia (AML) RNA samples. The intensities of Affymetrix array probe sets in three mapping groups A, B, and C are separately compared to the corresponding RNA-Seq gene counts in panels (a), (b), and (c) for one of the 175 acute myeloid leukemia (AML) RNA samples from The Cancer Genome Atlas (TCGA) acute myeloid leukemia study. The microarray data are MAS5 normalized and the RNA-Seq data are scaled as RPKM. The mappings from array probe sets to RNA-Seq genes are based on the gene ID mapping approach. [file 13059_2014_523_MOESM3_ESM.doc]

## Figure S3. The consistency of Affymetrix HG-U133_Plus_2 microarray and RNA-Seq gene expression levels for the acute myeloid leukemia (AML) RNA samples.

The intensities of Affymetrix array probe sets in three mapping groups A, B, and C are separately compared to the corresponding RNA-Seq gene counts in panels **(a)**, **(b)**, and **(c)** for one of the 175 acute myeloid leukemia (AML) RNA samples from The Cancer Genome Atlas (TCGA) acute myeloid leukemia study. The microarray data are MAS5 normalized and the RNA-Seq data are scaled as RPKM. The mappings from array probe sets to RNA-Seq genes are based on the gene ID mapping approach.
