# Supplementary material for: An investigation of biomarkers derived from legacy microarray data for their utility in the RNA-seq era
Source: Genome Biol. 2014 Dec 3;15(12):3273. doi: 10.1186/s13059-014-0523-y (PMC4290828; doi:10.1186/s13059-014-0523-y)
Supplement: Additional file 4: Figure S4. — The consistency of Agilent microarray and RNA-Seq gene expression levels for human RNA samples. The intensities of Agilent array probes in three mapping groups A, B, and C are separately compared to the corresponding RNA-Seq gene counts in panels (a), (b), and (c) for one of the 498 neuroblastoma RNA samples from the FDA SEquencing Quality Control (SEQC) project. The mappings from Agilent probes to RNA-Seq genes are based on the gene ID mapping approach. The microarray data are from Agilent customized 4 × 44 K oligonucleotide arrays, and RNA-Seq reads are from Illumina HiSeq 2000 with gene counts from the P2 pipeline (Novoalign with RefSeq human gene models). [file 13059_2014_523_MOESM4_ESM.doc]

## Figure S4. The consistency of Agilent microarray and RNA-Seq gene expression levels for human RNA samples.

The intensities of Agilent array probes in three mapping groups A, B, and C are separately compared to the corresponding RNA-Seq gene counts in panels **(a)**, **(b)**, and **(c)** for one of the 498 neuroblastoma RNA samples from the FDA SEquencing Quality Control (SEQC) project. The mappings from Agilent probes to RNA-Seq genes are based on the gene ID mapping approach. The microarray data are from Agilent customized 4×44K oligonucleotide arrays, and RNA-Seq reads are from Illumina HiSeq 2000 with gene counts from the P2 pipeline (Novoalign with RefSeq human gene models).
