# Supplementary material for: An investigation of biomarkers derived from legacy microarray data for their utility in the RNA-seq era
Source: Genome Biol. 2014 Dec 3;15(12):3273. doi: 10.1186/s13059-014-0523-y (PMC4290828; doi:10.1186/s13059-014-0523-y)
Supplement: Additional file 5: Figure S5. — The consistency of microarray gene expression levels and six sets of RNA-Seq gene counts derived from the same set of RNA-Seq raw data but using a diversity of RNA-Seq data analysis approaches. The MAS5 normalized microarray gene expression levels of mapping groups A, B, and C are plotted against the corresponding RNA-Seq measurements generated by six independent data analysis teams with a variety of bioinformatics pipelines and references, that is, (a) P1 (NCBI Magic), (b) P2 (Novoalign with RefSeq rat gene models), (c) P3 (BWA + RefSeq Rat RNAs), (d) P4 (Tophat + HTSeq with RefSeq rat gene models), (e) P5 (Bowtie + RSEM with Ensembl rat gene models), and (f) P6 (Tophat + Cufflinks de novo assembly). The mappings from microarrays to P1, P2, P3, and P4 gene sets are based on the gene ID mapping approach, while to P5 and P6 gene sets on the genome location mapping. The data set containing 62 rat liver RNAs is from sample profiling in the FDA SEquencing Quality Control toxicogenomics study with separate assays for each individual RNA sample from Affymetrix Rat_230_2 arrays and Illumina HiScanSQ/HiSeq 2000 RNA-Seq. In each of the six subpanels, gene expression measurements for mapping groups A, B, and C from microarrays are plotted against those from RNA-Seq in scatter plots (1), (2), and (3), respectively. [file 13059_2014_523_MOESM5_ESM.doc]

## Figure S5. The consistency of microarray gene expression levels and six sets of RNA-Seq gene counts derived from the same set of RNA-Seq raw data but using a diversity of RNA-Seq data analysis approaches.

The MAS5 normalized microarray gene expression levels of mapping groups A, B, and C are plotted against the corresponding RNA-Seq measurements generated by six independent data analysis teams with a variety of bioinformatics pipelines and references, that is, **(a)** P1 (NCBI Magic), **(b)** P2 (Novoalign with RefSeq rat gene models), **(c)** P3 (BWA + RefSeq Rat RNAs), **(d)** P4 (Tophat + HTSeq with RefSeq rat gene models), **(e)** P5 (Bowtie + RSEM with Ensembl rat gene models), and **(f)** P6 (Tophat + Cufflinks *de novo* assembly). The mappings from microarrays to P1, P2, P3, and P4 gene sets are based on the gene ID mapping approach, while to P5 and P6 gene sets on the genome location mapping. The data set containing 62 rat liver RNAs is from sample profiling in the FDA SEquencing Quality Control toxicogenomics study with separate assays for each individual RNA sample from Affymetrix Rat_230_2 arrays and Illumina HiScanSQ/HiSeq 2000 RNA-Seq. In each of the six subpanels, gene expression measurements for mapping groups A, B, and C from microarrays are plotted against those from RNA-Seq in scatter plots (1), (2), and (3), respectively.
