# Supplementary material for: An investigation of biomarkers derived from legacy microarray data for their utility in the RNA-seq era
Source: Genome Biol. 2014 Dec 3;15(12):3273. doi: 10.1186/s13059-014-0523-y (PMC4290828; doi:10.1186/s13059-014-0523-y)
Supplement: Additional file 7: Figure S7. — A performance comparison of support vector machine (SVM) models and their corresponding transferred models based on the SEQC NB data. For each of the six binary clinical endpoints and each of the three mapping groups A, B, and C, a set of 500 SVM models were developed from microarray training data and used to predict microarray validation samples. The signature genes of each of the 500 models were then used with all RNA-Seq training data for those genes to build a RNA-Seq model to predict RNA-Seq validation samples. Finally, the average prediction accuracies of the 500 microarray models are plotted against those of the 500 corresponding RNA-Seq models (a), with the per sample agreement better than chance evaluated with the Kappa statistic as shown in (b). The transferability of the signature genes from RNA-Seq back to microarray data was conversely computed. The 500 SVM models trained from RNA-Seq data were used to predict RNA-Seq validation samples. Then the signature genes of each RNA-Seq model were used with all microarray training data for those genes to build a microarray model to predict microarray validation samples. The average accuracies of the 500 RNA-Seq models were then compared to those of the 500 corresponding microarray models (c), with the per sample agreement better than chance assessed with the Kappa statistic as shown in (d). The six symbols in each panel represent the six binary clinical endpoints with green, blue, and orange colors denoting mapping groups A, B, and C, respectively. In panels (b) and (d), each symbol denotes the average Kappa statistic of the 500 pairs of models; and each error bar shows the 95% confidence interval (CI) for the mean Kappa statistic. Each CI was calculated with the bootstrap estimation. No significant difference is observed between trained microarray models and transferred untrained RNA-Seq models (paired t-test P is 0.557) and between trained RNA-Seq models and transferred untrained microarray models (paired t-te [file 13059_2014_523_MOESM7_ESM.doc]

## Figure S7. A performance comparison of support vector machine (SVM) models and their corresponding transferred models based on the SEQC NB data.

For each of the six binary clinical endpoints and each of the three mapping groups A, B, and C, a set of 500 SVM models were developed from microarray training data and used to predict microarray validation samples. The signature genes of each model were then used with all RNA-Seq training data for those genes to build a RNA-Seq model to predict RNA-Seq validation samples. Finally, the average prediction accuracies of microarray models are plotted against those of corresponding RNA-Seq models **(a)**, with the per sample agreement better than chance evaluated with the Kappa statistic as shown in **(b)**. The transferability of the signature genes from RNA-Seq back to microarray data was conversely computed. The 500 SVM models trained from RNA-Seq data were used to predict RNA-Seq validation samples. Then the signature genes of each RNA-Seq model were used with all microarray training data for those genes to build a microarray model to predict microarray validation samples. The average accuracies of RNA-Seq models were then compared to those of corresponding microarray models **(c)**, with the per sample agreement better than chance assessed with the Kappa statistic as shown in **(d)**. The six symbols in each panel represent the six binary clinical endpoints with green, blue, and orange colors denoting mapping groups A, B, and C, respectively. In panels (b) and (d), each symbol denotes the average Kappa statistic of the 500 pairs of models; and each error bar shows the 95% confidence interval (CI) for the mean Kappa statistic. Each CI was calculated with the bootstrap estimation. No significant difference is observed between trained microarray models and transferred untrained RNA-Seq models (paired t-test *P* is 0.557) and between trained RNA-Seq models and transferred untrained microarray models (paired t-test *P* is 0.158).
