# Supplementary material for: An investigation of biomarkers derived from legacy microarray data for their utility in the RNA-seq era
Source: Genome Biol. 2014 Dec 3;15(12):3273. doi: 10.1186/s13059-014-0523-y (PMC4290828; doi:10.1186/s13059-014-0523-y)
Supplement: Additional file 8: Table S1. — Performance metrics for the assessment of cross-platform transferability of signature genes of NSC models based on the SEQC NB data. [file 13059_2014_523_MOESM8_ESM.doc]

## Table S1. Performance metrics for the assessment of cross-platform transferability of signature genes of NSC models based on the SEQC NB data.

A*: A_EFS_All; B*: B_OS_All; C*: C_SEX_All; D*: D_FAV_All; E*: E_EFS_HR; F*: F_OS_HR; AUC: Area under ROC curve; CI: Confidence interval; 95% CI was calculated from the bootstrap estimation. The upper-right and lower-left regions in gray are for the untrained models built using cross-platform transferred signature genes, while the upper-left and lower-right regions are for the models originally trained.
