# Supplementary material for: An investigation of biomarkers derived from legacy microarray data for their utility in the RNA-seq era
Source: Genome Biol. 2014 Dec 3;15(12):3273. doi: 10.1186/s13059-014-0523-y (PMC4290828; doi:10.1186/s13059-014-0523-y)
Supplement: Additional file 15: Figure S11. — A performance comparison of nearest shrunken centroids (NSC) models in predicting microarray and RNA-Seq validation samples, based on the SEQC NB Data without per sample z-score transformation. In the comparison, microarray and RNA-Seq data were log2 intensity data and log2 counts, respectively. For each of the six binary clinical endpoints and each of the two mapping groups A and B, a set of 500 NSC models were developed from microarray and RNA-Seq training data independently. Each set of NCS models were then used to predict both microarray and RNA-Seq validation samples. The average prediction accuracies of the 500 microarray-based models in prediction microarray data were plotted against those in predicting RNA-Seq data (a), with per sample agreement better than chance assessed with the Kappa statistic as shown in (b); while the average accuracies of the 500 RNA-Seq-based models in predicting RNA-Seq data were compared to those in predicting microarray data (c), with per sample agreement better than chance evaluated with the Kappa statistic as shown in (d). The six symbols in each panel represent the six binary clinical endpoints with green and blue colors denoting mapping groups A and B, respectively. In panels (b) and (d), each symbol denotes the average Kappa statistic of 500 pairs of prediction results; and each error bar shows the 95% confidence interval (CI) for the mean Kappa statistic. Each CI was calculated with the bootstrap estimation. [file 13059_2014_523_MOESM15_ESM.doc]

## Figure S11. A performance comparison of nearest shrunken centroids (NSC) models in predicting microarray and RNA-Seq validation samples, based on the SEQC NB Data without per sample z-score transformation.

In the comparison, microarray and RNA-Seq data were log_2_ intensity data and log_2_ counts, respectively. For each of the six binary clinical endpoints and each of the two mapping groups A and B, a set of 500 NSC models were developed from microarray and RNA-Seq training data independently. Each set of NCS models were then used to predict both microarray and RNA-Seq validation samples. The average prediction accuracies of the 500 microarray-based models in prediction microarray data were plotted against those in predicting RNA-Seq data **(a)**, with per sample agreement better than chance assessed with the Kappa statistic as shown in **(b)**; while the average accuracies of the 500 RNA-Seq-based models in predicting RNA-Seq data were compared to those in predicting microarray data **(c)**, with per sample agreement better than chance evaluated with the Kappa statistic as shown in **(d)**. The six symbols in each panel represent the six binary clinical endpoints with green and blue colors denoting mapping groups A and B, respectively. In panels (b) and (d), each symbol denotes the average Kappa statistic of 500 pairs of prediction results; and each error bar shows the 95% confidence interval (CI) for the mean Kappa statistic. Each CI was calculated with the bootstrap estimation.
