# Supplementary material for: An investigation of biomarkers derived from legacy microarray data for their utility in the RNA-seq era
Source: Genome Biol. 2014 Dec 3;15(12):3273. doi: 10.1186/s13059-014-0523-y (PMC4290828; doi:10.1186/s13059-014-0523-y)
Supplement: Additional file 17: Table S5. — Concordance indices and P values for the assessment of cross-platform transferability of signature genes of Cox models based on the SEQC NB data. [file 13059_2014_523_MOESM17_ESM.doc]

## Table S5. Concordance indices and *P* values for the assessment of cross-platform transferability of signature genes of Cox models based on the SEQC NB data.

A*: A_EFS_All; B*: B_OS_All; E*: E_EFS_HR; F*: F_EFS_HR; C.Index: Concordance index calculated with R package survcomp [27], CI: Confidence interval; p1, p2, and p3 are *P* values indicating whether a C.Index is significantly different from 0.5; p4 is a *P* value denoting whether the concordance index of the original trained Cox model is significantly greater than that of the corresponding Cox model built using transferred gene signatures. The columns in Leave-one-out training contain the performance estimation for leave-one-out cross-validation process.
