# Supplementary material for: An investigation of biomarkers derived from legacy microarray data for their utility in the RNA-seq era
Source: Genome Biol. 2014 Dec 3;15(12):3273. doi: 10.1186/s13059-014-0523-y (PMC4290828; doi:10.1186/s13059-014-0523-y)
Supplement: Additional file 18: Table S6. — The performance of Cox proportional hazards models developed from one-platform in predicting microarray and RNA-Seq validation samples based on the SEQC NB data. [file 13059_2014_523_MOESM18_ESM.doc]

## Table S6. The performance of Cox proportional hazards models developed from one-platform in predicting microarray and RNA-Seq validation samples based on the SEQC NB data.

A*: A_EFS_All; B*: B_OS_All; E*: E_EFS_HR; F*: F_EFS_HR; C.Index: Concordance index calculated with R package survcomp [27], CI: Confidence interval; p1, p2, and p3 are *P* values indicating whether a C.Index is significant different from 0.5; p4 is a *P* value denoting whether the concordance index of a Cox model in predicting samples profiled with the same platform used for model development is significantly greater than that in predicting samples profiled with the other platform. The columns in Leave-one-out training contain the performance estimation for leave-one-out cross-validation process.
