# Supplementary material for: An investigation of biomarkers derived from legacy microarray data for their utility in the RNA-seq era
Source: Genome Biol. 2014 Dec 3;15(12):3273. doi: 10.1186/s13059-014-0523-y (PMC4290828; doi:10.1186/s13059-014-0523-y)
Supplement: Additional file 19: Figure S13. — A performance comparison of nearest shrunken centroids (NSC) models and their corresponding transferred models based on the TCGA AML data. For each of the two binary clinical endpoints and each of the three mapping groups A, B, and C, a set of 500 NSC models were developed from microarray training data and used to predict microarray validation samples. The signature genes of each of the 500 microarray models were then used with all RNA-Seq training data for those genes to build an untrained RNA-Seq model to predict RNA-Seq validation samples. Finally, the average prediction accuracies of the 500 microarray models are plotted against those of the 500 corresponding RNA-Seq models (a), with the per sample agreement better than chance evaluated with the Kappa statistic as shown in (b). The transferability of the signature genes from RNA-Seq back to microarray data was conversely calculated. The 500 NSC models trained from RNA-Seq data were used to predict RNA-Seq validation samples. Then the signature genes of each RNA-Seq model were used with all microarray training data for those genes to build an untrained NSC model to predict microarray validation samples. The average accuracies of the 500 RNA-Seq models were then compared to those of the 500 corresponding microarray models (c), with the per sample agreement better than chance assessed with the Kappa statistic as shown in (d). The two symbols in each panel represent the two binary clinical endpoints with green, blue, and orange colors denoting mapping groups A, B, and C, respectively. In panels (b) and (d), each symbol denotes the average Kappa statistic of the 500 pairs of model predictions; and each error bar shows the 95% confidence interval (CI) for the mean Kappa statistic. Each CI was calculated with the bootstrap estimation. No significant difference is observed between trained microarrays models and transferred RNA-Seq models (paired t-test P is 0.354) and between the trained RNA-Seq models and the transferr [file 13059_2014_523_MOESM19_ESM.doc]

## Figure S13. A performance comparison of nearest shrunken centroids (NSC) models and their corresponding transferred models based on the TCGA AML data.

For each of the two binary clinical endpoints and each of the three mapping groups A, B, and C, a set of 500 NSC models were developed from microarray training data and used to predict microarray validation samples. The signature genes of each microarray model were then used with all RNA-Seq training data for those genes to build an untrained RNA-Seq model to predict RNA-Seq validation samples. Finally, the average prediction accuracies of microarray models are plotted against those of corresponding RNA-Seq models **(a)**, with the per sample agreement better than chance evaluated with the Kappa statistic as shown in **(b)**. The transferability of the signature genes from RNA-Seq back to microarray data was conversely calculated. The 500 NSC models trained from RNA-Seq data were used to predict RNA-Seq validation samples. Then the signature genes of each RNA-Seq model were used with all microarray training data for those genes to build an untrained NSC model to predict microarray validation samples. The average accuracies of RNA-Seq models were then compared to those of corresponding microarray models **(c)**, with the per sample agreement better than chance assessed with the Kappa statistic as shown in **(d)**. The two symbols in each panel represent the two binary clinical endpoints with green, blue, and orange colors denoting mapping groups A, B, and C, respectively. In panels (b) and (d), each symbol denotes the average Kappa statistic of the 500 pairs of model predictions; and each error bar shows the 95% confidence interval (CI) for the mean Kappa statistic. Each CI was calculated with the bootstrap estimation. No significant difference is observed between trained microarrays models and transferred RNA-Seq models (paired t-test *P* is 0.354) and between the trained RNA-Seq models and the transferred microarray models (paired t-test *P* is 0.106).
